# Protocol to evaluate a pilot program to upskill clinicians in providing genetic testing for familial melanoma

**DOI:** 10.1371/journal.pone.0275926

**Published:** 2022-12-07

**Authors:** Clare A. Primiero, Anna Finnane, Tatiane Yanes, Betsy Peach, H. Peter Soyer, Aideen M. McInerney-Leo

**Affiliations:** 1 The University of Queensland Diamantina Institute, The University of Queensland, Dermatology Research Centre, Brisbane, Australia; 2 The University of Queensland, School of Public Health, Brisbane, Australia; 3 Department of Dermatology, Princess Alexandra Hospital, Brisbane, QLD, Australia; Prince Sattam Bin Abdulaziz University, College of Applied Medical Sciences, SAUDI ARABIA

## Abstract

**Introduction:**

Genetic testing for hereditary cancers can improve long-term health outcomes through identifying high-risk individuals and facilitating targeted prevention and screening/surveillance. The rising demand for genetic testing exceeds the clinical genetic workforce capacity. Therefore, non-genetic specialists need to be empowered to offer genetic testing. However, it is unknown whether patient outcomes differ depending on whether genetic testing is offered by a genetics specialist or a trained non-genetics clinician. This paper describes a protocol for upskilling non-genetics clinicians to provide genetic testing, randomise high-risk individuals to receive testing from a trained clinician or a genetic counsellor, and then determine whether patient outcomes differed depending on provider-type.

**Methods:**

An experiential training program to upskill dermatologically-trained clinicians to offer genetic testing for familial melanoma is being piloted on 10–15 clinicians, prior to wider implementation. Training involves a workshop, comprised of a didactic learning presentation, case studies, simulated sessions, and provision of supporting documentation. Clinicians later observe a genetic counsellor led consultation before being observed leading a consultation. Both sessions are followed by debriefing with a genetic counsellor. Thereafter, clinicians independently offer genetic testing in the clinical trial.

Individuals with a strong personal and/or family history of melanoma are recruited to a parallel-group trial and allocated to receive pre- and post- genetic testing consultation from a genetic counsellor, or a dermatologically-trained clinician. A mixed method approach measures psychosocial and behavioural outcomes. Longitudinal online surveys are administered at five timepoints from baseline to one year post-test disclosure. Semi-structured interviews with both patients and clinicians are qualitatively analysed.

**Significance:**

This is the first program to upskill dermatologically-trained clinicians to provide genetic testing for familial melanoma. This protocol describes the first clinical trial to compare patient-reported outcomes of genetic testing based on provider type (genetic counsellors vs trained non-genetic clinicians).

## Introduction

The average lifetime risk of cutaneous melanoma in Australia is 5% [1], however individuals carrying high penetrance pathogenic germline variants in genes such as *CDKN2A*, incur a lifetime risk of 52–80% [2–4]. First degree relatives of mutation carriers hold a 50:50 chance of inheriting the same pathogenic mutation, due to the autosomal dominant pattern of inheritance [5]. Recent systematic reviews on the impact of genetic testing for familial melanoma reported positive outcomes for protective behaviour [6] without causing psychological harm [7]. However, the evidence produced to date has yet to be translated into routine clinical care. In Australia, which has the highest global incidence of melanoma, it is predicted that 5–10% of individuals with melanoma would meet genetic testing eligibility criteria [8].

Genomics is increasingly being incorporated into clinical care to identify high-risk individuals and customise treatment [9]. Currently, pre- and post-test counselling provided by genetic counsellors is considered the ‘gold standard’ method for providing genetic testing. However, as genetic technology, such as Next Generation Sequencing advances, and associated costs decrease, there is an expected shortfall of the clinical genetic workforce to meet demand [9–11]. Upskilling non-genetic specialists to provide genetic testing, in a process known as mainstreaming, has increasingly been implemented in oncology care, specifically for breast and ovarian cancer [11,12]. Upskilling clinicians to provide genomic testing into routine care is feasible when minimal risk of psychological distress is predicted and when the pattern of inheritance and impact on disease risk are well understood [13,14]. Previous mainstreaming interventions have reported significant advantages, such as decreased healthcare costs [15–17] and waiting times [15,16,18–22], while increasing access to genomic services and identification of pathogenic mutation carriers [18,23].

A recent cross-sectional survey on Australian Dermatologists provides insight into the current use and attitudes towards genetic testing in dermatological practice [24]. This study reported that genetic testing was considered highly relevant to future dermatological practice and showed a demand for resources to upskill in this area. Comparable studies, surveying dermatology fellowship program directors [25], paediatric dermatologists [26], and trainees [27] have reported a gap in genomic medicine education and a recognition of relevance to their practice. Familial melanoma genetic testing is an ideal genetic test to mainstream into dermatologist clinics given the positive psychological and behavioural outcomes reported from previous studies [28,29], the affordability of the test, and with appropriate education, the ease of test ordering and result interpretation.

This paper describes the protocol for a trial to evaluate whether patient psycho-behavioural outcomes and satisfaction with the genetic testing process is impacted by provider type. The authors have developed a training program for providing genetic testing for familial melanoma and are piloting the program in a small cohort of dermatologically-trained clinicians (referred to throughout as ‘clinicians’, as distinct from ‘genetic counsellors’) prior to wider clinical implementation. This study uses mixed methods for data collection of both quantitative and qualitative outcomes from both consumers of genetic testing, and healthcare providers. Longitudinal, validated instruments are used to analyse patient reported outcomes. Qualitative methods are employed to describe patient’s experience of living with familial melanoma and the genetic testing process. Interviews with clinicians participating in a training program provides constructive feedback to guide a future training initiative for clinical implementation.

## Methods

### Research question

The clinical question addressed in the trial follows a PICO model (Patient, Intervention, Comparator and Outcomes) [30]. In this study, the *patients* are people with significant personal and family history of melanoma, indicative of familial melanoma [31]. The *intervention* is receiving pre- and post-test genetic consultation from a non-genetic clinician trained in our pilot program. The *comparator* is receiving pre- and post-test genetic consultation from a qualified genetic counsellor, which is the current standard of care in Australia. The primary *outcome* is patient satisfaction with the genetic testing process, measured using the validated Genetic Counselling Satisfaction Scale (GCSS) [32] two weeks after test results are disclosed to patients. Secondary outcomes include patients’ psychosocial well-being, health beliefs, and preventative behaviour, measured using validated instruments at multiple timepoints.

### Trial design

This protocol has been prepared in concordance with the Standard Protocol Items Recommendations for Interventional Trials (SPIRIT) statement [33]. The complete SPIRIT checklist of items addressed is available in (S1 Fig).

### Patient/Public involvement statement

This research is being conducted by The University of Queensland’s Dermatology Research Centre (UQ-DRC). The UQ-DRC have been working closely with members of the public regarding skin surveillance research since 2010. The UQ-DRC have been conducting biannual consumer forums since 2016, to both inform the general public and research patients on our research progress, and to also provide a platform for consumer engagement.

### Intervention

This protocol describes a longitudinal parallel group clinical trial, with quasi-experimental allocation using a 1:1 ratio. While the investigators have no influence in the group assignment of patients, patients are assigned to receive their pre- and post-test genetic testing consultation by either a genetic counsellor or a clinician trained in the study to deliver genetic testing. Assignment is based on patient and provider availability, which reflects the real-world clinical setting, as provider and patient availability is unpredictable.

### Clinician training program

Participation in the clinician training program is offered to clinicians currently involved in research with the UQ-DRC. This cohort of clinicians include both experienced dermatologists, trainee dermatologists and dermatologically-trained clinicians. No minimum years of medical experience were required, nor are there other eligibility criteria for clinician involvement. This study aims to train between 10–15 clinicians to provide genetic testing in the clinical trial.

Development of the training program for clinicians to provide genetic testing for familial melanoma predominantly follows the guiding principles described by Kolb’s Experiential learning model [34]. This model describes a four-staged learning cycle involving concrete experiences, reflective observation, abstract conceptualisation, and active experimentation [34]. The training program has been developed by authors AMM-L and BP whom are qualified genetic counsellors, and author CAP an experienced clinical research coordinator. The program comprises of an initial two-hour workshop composed of a brief didactic presentation, case-based learning, and mock genetic education consultations for familial melanoma. Resources, including a counselling-aid flipbook to use during patient consultations, and standard operating procedures describing the genetic testing consultation in detail, are provided for clinicians. The workshop covers multiple domains important to providing genetic testing, including identifying individuals eligible for genetic testing, recording family history in a pedigree and risk stratification of family members, reviewing the genetics of hereditary melanoma, discussing strengths and limitations of the testing including the impact on insurance and current protections in place, preparing consumers for possible results and most likely outcomes, ordering genetic testing, interpreting and discussing the test results, and, referring to clinical genetic services when applicable.

After completing the workshop, clinicians observe a genetic counsellor conduct a consultation for familial melanoma, after which they lead their own consultation with a genetic counsellor observing. After both appointments the clinician reflects on the session with the genetic counsellor and any queries are answered. From then on, the trained clinician independently leads genetic testing consultations for familial melanoma within the parallel group trial. The process is repeated for the post-test disclosure session. All molecular results are discussed and reviewed in a multi-disciplinary team meeting to ensure consistency in result interpretation and upskill dermatologically trained clinicians in interpreting and explaining genetic test results.

### Outcomes

The primary outcome is patient satisfaction with the genetic testing process, measured using the GCSS [32], completed two weeks after genetic test result disclosure. Secondary objectives are to report on patient’s long-term psychological impact of genetic testing, as well as any impact on personal empowerment and engaging in protective behaviours. Qualitative methods are used to explore the feasibility, as well as the experience of delivering and receiving genetic testing, from the perspective of both consumers and healthcare providers. Interviews with study patients, one month after test result disclosure explore the cognitive and social impact of living with familial melanoma, in addition to the impact of genetic testing. Interviews with clinicians trained to provide genetic testing collects feedback on the training program, and perceptions on the usefulness of genetic testing for familial melanoma.

### Flow of study participants

**Fig 1:**
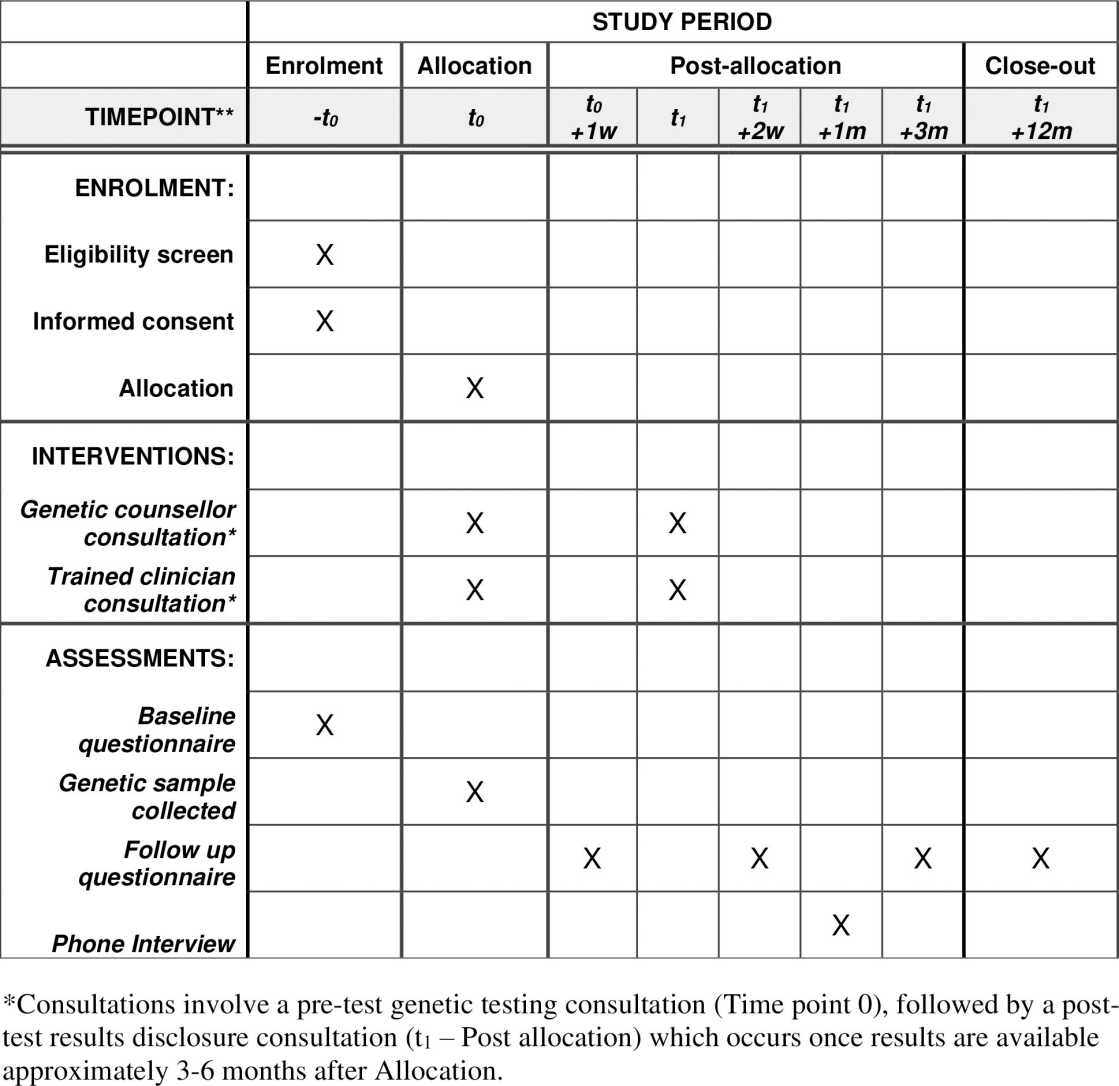
Standard protocol time points and items based on the SPIRIT schedule of enrolment, interventions, and assessments.

The flow of study procedures is illustrated using the SPIRIT schedule of enrolment, interventions, and assessments in Fig 1. The pilot trial involves two appointments for each participant, which are completed in-person at the Clinical Research Facility, at the Princess Alexandra Hospital in Brisbane, Australia, or conducted using video teleconferencing software, such as Zoom. A previous systematic review reported that patient satisfaction with tele-genetic services was high [35], and therefore is included as an option in response to the COVID-19 pandemic restrictions.

The first baseline appointment (the ‘Pre-test appointment’) involves recording informed consent, completing online baseline questionnaire, pre-test genetic education and counselling (provided by either a trained clinician or genetic counsellor), and collection of a saliva sample. Participants are made aware (unblinded) of the qualifications of the provider leading their appointments. Visits take between 1 to 1.5 hours. The second appointment (the ‘test reporting appointment’) takes place approximately 3 months later, when genetic test results are available. The same provider, or provider type conducts the pre and post-test consultations. Any changes of provider are recorded, to allow examination of any impact on participant experience. Genetic test results are reported, using a standard protocol. If applicable, the research team discusses with the participant which family members to invite to the research study.

One month after participants have received their genetic test results, they are asked to participate in a phone interview to discuss their experiences with melanoma in the family, and experiences with genetic testing. Interviews are conducted in-person or over the phone, and audio taped, then transcribed verbatim by a professional transcription service.

### Sample size

This trial is designed as a non-inferiority study to consider whether genetic testing performed by trained clinicians is as satisfactory to participants as testing performed by genetic counsellors. The primary outcome is patient satisfaction measured on a 30-point scale (GCSS). Based on a non-inferiority limit of a two-point difference and using the previously reported standard deviation on GCSS in a cancer population of 3.87, sample size calculations were performed. Assuming type I error rate of 95% and 80% power, the required sample size is 46 participants per arm.

### Eligibility criteria

People at high risk of familial melanoma are identified using the GenoMELPREDICT tool, which is designed to predict *CDKN2A* mutational status in people from melanoma prone families [31]. The tool considers age of first melanoma diagnosis, number of primary melanomas, number of family members diagnosed with melanoma, and whether any family members were previously diagnosed with pancreatic cancer (also associated with CDKN2A mutation). Individuals with ≥10% probability of carrying a *CDKN2A* mutation, as calculated by the GenoMELPREDICT tool, are eligible to participate in the study.

### Recruitment

The University of Queensland’s Dermatology Research Centre (UQ-DRC) maintains a database of volunteers interested in skin surveillance research, including those with varying risk of melanoma. This database is used to identify individuals with personal and family history indicative of familial melanoma who are contacted to invite to participate in the current study. Furthermore, the UQ-DRC receives ongoing referrals from local (South-East Queensland, Australia) Dermatologists and Skin Examination Centres for high-risk patients interested in participating in research. These prospective referrals are screened for eligibility. Eligible potential participants are initially contacted by phone to gauge interest in the study, confirm eligibility, and if receptive, a Participant Information and Consent Form (PICF) is emailed to them. They are encouraged to read over and discuss the PICF with family members and healthcare providers. A follow up call approximately two weeks later answers any questions and schedules a baseline appointment for those willing to participate.

Family members of participants who receive a positive genetic test results are invited to participate in the study (cascade testing). Segregation analysis is offered to family members of individuals who receive variants of unknown significance, to assess whether the variant segregates appropriately with affection status. Recruitment of family members is first discussed with the proband (i.e., the first individual to have genetic testing), who then initiates discussions with their at-risk family members. The proband is provided with copies of the PICF to share with family members, including the contact details of the study team. If family members express an interested in participating in the pilot study, they are contacted by the study team.

### Data collection

#### Development of questionnaire

To achieve the research objectives, four distinct categories of outcomes are established to guide questionnaire development of the study.

1. Experience with genetic testing: How do patients rate their experience, are they satisfied, and is there any regret in learning genetic results? Are there any differences in these outcomes when testing is provided by a clinician as compared to a genetic counsellor?
2. Health beliefs: Does genetic testing influence personal risk perception or perceived control of future melanomas? Has testing changed or produced fatalistic beliefs?
3. Psychological well-being: Does genetic testing cause negative psychological impact such as anxiety or depression? If so, how does this vary over time or vary with provider type? Are there personality or demographic traits that are predictors for adverse outcomes?
4. Sun behaviour and screening: is there any change from baseline preventative behaviour after receiving genetic test results? How do these factors vary over time, by test result and by provider type?

Where possible, previously validated instruments are used to address the research questions, some of which modified slightly for applicability to familial melanoma. Table 1 provides details on each instrument selected for the questionnaires, included a description of its purpose, and at which questionnaire timepoints it is administrated.

**Table 1:** A list of the different instruments used to address research questions, across the five questionnaire timepoints.

Research Question	Validated Instrument	Questionnaire time points^#^				
		1	2	3	4	5
Patient’s experience	Genetic Counselling Satisfaction Scale (GCSS) A scale used to capture satisfaction with all aspects of genetic counselling and test reporting [32].		✓	✓		
	Genomic Outcomes Scale (GOS) A short 6-item version of the original GOS-24. Used to measure patient outcomes from genetic testing and counselling services [36].			✓		
	Perceived benefits/limitations A scale used to measure personal beliefs about the benefits and limitations of genetic testing that can influence decisions about accepting/declining genetic testing [37].	✓				
	Decision regret scale A scale used to measure any regret in a patient’s healthcare decisions [38].			✓		✓
	Decision satisfaction A scale used to capture the different aspects of satisfaction in healthcare decisions [39].			✓		
	Multidimensional Impact of Cancer Risk Assessment (MICRA) A tool designed specifically to measure the impact of genetic testing. It includes 3 subscales to measure distress, uncertainty, and positive experiences [40].			✓	✓	✓
Perceptions of risk and control (Individual beliefs)	Risk perception Questions used by Kasparian (2009), based on previous research in both breast and skin cancer, to measure perceptions on susceptibility compared to general population and perceived control [37].	✓	✓	✓	✓	✓
	Health Belief Model Survey Survey for personal perceptions of susceptibility, severity, combined with benefits and barriers of preventative behaviour. Adapted to be melanoma specific [41,42].	✓				✓
	Multidimensional Health Locus of Control (MHLC) Scale A widely used scale to predict health behaviour. Uses personal beliefs on self-responsibility (internal), powerful others (e.g., healthcare providers), and the role of chance, luck or fate plays in health outcomes [43,44].	✓				
	Perceived Personal Control (PPC) Scale PPC is recognised as an important outcome of genomic testing, as a variable for coping with a health threat [45].	✓				✓
	Fatalism Scale A scale to measure personal beliefs about the role of luck or fate has when facing a serious health problem [46].	✓			✓	✓
	Belief in genetic determinism Scale A sub-scale of the PUGGS (Public Understanding and attitudes towards Genetics and Genomics), used to measure perceptions on how genetic and environmental factors contribute to traits [47].	✓				
Psychological well-being (Modifying factors	Hospital Anxiety and Depression Scale (HADS) Developed in 1983 and most widely used and reliable instrument to measure incidence of anxiety and depression [48].	✓	✓	✓	✓	✓
	Cancer Worry Scale A well-used scale to measure fear of cancer recurrence and worry about the risk of developing cancer (again) [49].	✓			✓	✓
	Monitor/blunter Scale (abbreviated) An abbreviated 2-item version to assess whether individuals seek out (monitor) or avoid (blunter) information when faced with threatening events [50,51].		✓			
	State-Trait Anxiety Inventory (STAI) A shortened 6-item version, based on original 40-item instrument. Used to measure anxiety as both a state and trait in an individual [52].		✓			
Health behaviour (Action)	Sun exposure Past and present sun exposure, including occupational and recreational. Sun protective behaviour currently followed [53,54].	✓			✓	✓
	Skin surveillance Frequency of clinical and self-skin examinations [53,54].	✓			✓	✓

**#** Questionnaires are listed numerically and represent the following timepoints: 1) baseline (prior to pre-test consultation appointment), 2) One week after pre-test consultation appointment, 3) Two weeks after test result disclosure appointment, 4) Three months after test result disclosure, and 5) Twelve months after test result disclosure. Average time required to complete questionnaires ranges from approximately 30 minutes for baseline questionnaire, to 10 minutes for questionnaire two.

**Fig 2:**
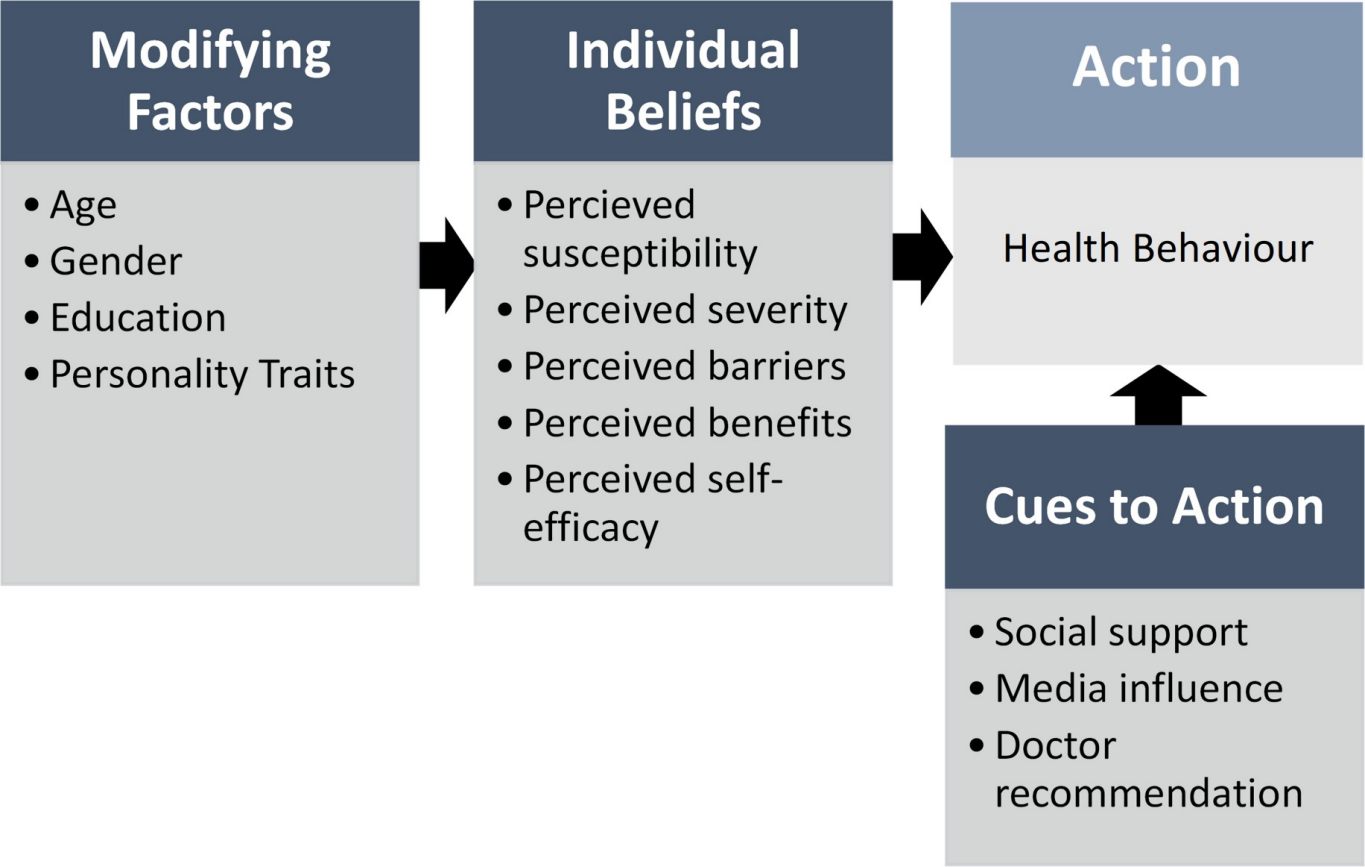
An overview of variables included in the Health Belief Model, used to predict uptake of a health behaviour.

When selecting instruments to measure health beliefs and psychological well-being, a theoretical framework was selected to guide questionnaire development. The Health Belief Model (HBM), provides a paradigm for explaining protective behaviour when faced with a health threat [41]. Previous studies have applied the HBM model for prediction of sun protective behaviours [55–57], and uptake of predictive genetic testing [58–60]. The HBM model considers three categories of variables that can impact compliance with a recommended health behaviour and is illustrated in Fig 2. The first category titled ‘Modifying Factors’ includes demographic and personality traits. The second category, ‘Individual Beliefs’ considers cognitive perceptions on the severity and susceptibility of a health threat, perceived benefits and barriers to protective health behaviour, and perceived self-efficacy in performing the health behaviour. The ‘Cues to Action’ category includes social support, media influence and doctor recommendations [61].

### Qualitative study design

The methodology, phenomenology is used to guide development of the qualitative component of this protocol. Phenomenology is the study of a phenomena, i.e., to understand the way different aspects of life are experienced from a subjective or first-person point of view [62]. Phenomenology is widely used to generate knowledge on the lived experiences of study participants, and often uses in-depth interviews to collect empirical data to describe and reconstruct events and social experiences [62].

Interviews are conducted with trained clinicians and participants who received their genetic testing result for familial melanoma. All interviews are informed by a semi-structed interview guide (S2 Fig). Participants interviews take place one month after genetic test results are disclosed, while clinicians are interviewed after they have independently provided genetic testing consultation and returned genetic test results at least once. The aim of the participant interview is to gain an insight into the lived experiences of day-to-day life with familial melanoma, and secondly, to understand the genetic testing process and impact from the participant’s perspective. The objectives of the clinician interview are to receive in-depth constructive feedback regarding the training program, and secondly, to record clinician’s perceptions on the usefulness and feasibility of genetic testing for familial melanoma. Interview questions and guides were developed based on a literature review.

### Genetic testing

Genetic testing is conducted by an accredited laboratory using a commercial familial melanoma gene panel (Invitae©). Participants are asked to sign the Invitae© consent form, if they wish to proceed with testing. The panel covers the following genes: *BAP1*, *BRCA1*, *BRCA2*, *CDK4*, *CDKN2A*, *MC1R*, *MITF*, *POLE*, *POT1*, *PTEN*, *RB1*, *TERT*, *and TP53*. The commercial testing can produce three possible results for each gene tested; negative, positive, or variant of uncertain significance (VUS). A copy of the results report is discussed and provided to the participants. Common red hair colour variants in the *MC1R* gene are also reported to participants, as they are known to be associated with increased risk of melanoma, and penetrance of CDKN2A variants [4,63].

### Data management

The clinical trial uses five online questionnaires, completed longitudinally by participants, comprised of previously validated scales. Table 1 details which scales are used, and at what timepoints. Questionnaires are managed using an online, locally administered application, REDCap (Research Electronic Data Capture). REDCap is highly secure and compliant with international standards for handling clinical data [64]. Participant data is stored confidentially, using a designated Study ID code in place of identifying information. Fig 1 illustrates the timeline of when online questionnaires are administered regarding clinical appointments. Participants are sent an email with a link to each questionnaire at the appropriate time, allowing completion directly into the REDCap study database. Two weekly email reminders are sent if the questionnaire is not completed.

Audio recordings of interview data are provided to a professional transcription service. Once interview transcripts are checked for accuracy, audio recordings are deleted. Both temporary audio files and interview transcript files are stored on The University of Queensland secured server and password protected. Genetic test results, and other appointment notes, are saved on the University’s secured server, with password protection.

### Data analysis plan

The primary outcome is the survey response to the GCSS at timepoint 3 (2 weeks after test results are disclosed). Secondary outcomes include patient’s long-term psychological impact of genetic testing, as well as any impact on engaging in protective behaviours, as captured using previously validated scales (described in Table 1). These will be scored and interpreted using methods as originally described by authors of the scales. Descriptive statistics will be used to summarise participant characteristics. Bivariate analyses will be conducted to examine clinically relevant differences in outcomes between groups, using crosstabulations and chi squared tests for categorical variables and t-tests for continuous variables. Specifically, differences in mean GCSS scores between the two participant groups (trained clinician versus genetic counsellor) will be compared using an unpaired t-test, with a p value <0.05 considered statistically significant. Logistic regression will be used to identify predictor psychosocial variables for GCSS. Analysis of secondary outcomes will be driven by the study aims and hypotheses to address whether participant reported outcomes and preventative behaviours differ depending on provider type for genetic testing consultation. Patterns of missing data will be examined, and where any variable has more than 10% missing data, a ‘missing’ category will be defined and included in analyses, to maximise the use of available data.

Thematic analysis will be used for qualitative analysis of interview transcripts, utilising NVivo Software to manage transcripts and the coding process. Thematic analysis is a widely used methodology to generate a description of key themes raised by interviewees and involves six core steps [65]. The methodology for analysis will follow methods described by Clark & Braun (2006) [66], and will include 1) familiarisation with transcripts, 2) Assigning codes to relevant or interesting text, 3) Recognise overarching themes that apply to connecting codes, 4) Review the themes and adjust coding where suitable, 5) Name and describe each theme, and 6) Generate a report for publication in the context of the research questions and current literature.

### Ethical and dissemination

This study has received Human Research Ethics Committee (HREC) approval received from both Metro South Health HREC (HREC/19/QMS/58196) and The University of Queensland HREC (#2019002762), and prospectively registered with the Australian New Zealand Clinical Trial Register (ANZCTR12620000043932). Any protocol modifications will be communicated to relevant parties. Study findings will be disseminated through peer-reviewed publications, conferences, and non-peer reviewed media outlets.

### Trial status and timeline

The study received Human Ethics approval in November 2019, and recruitment commenced March 2020. An HREC amendment was approved in March 2020 to use teleconferencing for patient appointments. Due to the Covid-19 global pandemic the study was put on temporary hold for several months before commencing again in August 2020. The study has faced multiple other appointment postponements in response to local outbreaks and restrictions. Recruitment is continuing through 2022/23.

## Discussion

This research addresses an important step towards meeting an emerging health challenge of integrating genomic medicine into routine clinical care. Mainstreaming genomic testing, by upskilling oncology specialists has already made a significant impact for participants with breast and ovarian cancer, through improving access to testing, reducing waiting periods, and significant healthcare cost savings [11,12]. The continuation of care possible through mainstreaming genetic testing, is highly valued by patients [15,67]. For familial melanoma, dermatologists are most ideally placed to recognise eligible patients, offer testing, and provide appropriate clinical management. Benefits of identifying pathogenic mutation carriers would extend to family members, as awareness is increased, and predictive genetic testing could identify younger, unaffected family members [68].

The successful development of an alternate health service delivery model requires engagement with stakeholders, including both consumers and healthcare providers to gauge receptiveness and perceived need. In conjunction with this research, the authors have recently performed a cross-sectional survey of Australian Dermatologists’ perceptions and use of genetic testing in practice [24]. The results from this study and a literature review provided evidence that genomic medicine is increasingly considered relevant to dermatological practice, while also reporting low levels of previous training and confidence in offering genetic testing. To date, mainstreaming interventions commonly use either no comparator or historical controls to evaluate efficacy. The current study uses quasi-experimental allocation in a parallel group design to compare the intervention to the current standard of care, to improve reliability of findings. The pilot training program for a genetic testing service delivery method, is tested against the current standard of care, to ensure that consumer outcomes (satisfaction, psychological well-being, and health behaviours) do not differ. Study findings will shape best practice guidelines for melanoma genetic testing in dermatology, recognising the potential to improve prevention and early diagnosis.

## Conclusion

To our knowledge, this is the first study to allocate participants to receive genetic testing from a genetic counsellor or a trained non-genetics clinician and determine whether provider type affects psychological and behavioural outcomes. The convergence of longitudinal quantitative participant reported outcomes, with qualitative insight from both participant consumers and from healthcare providers, provides both depth and breadth of understanding for delivery of genetic testing for familial melanoma. If findings support provision of genetic testing by clinicians, outcomes from this research will directly inform future training programs for dermatologists, facilitating the integration of genetic testing into mainstream dermatology clinics.

## Supporting information

S1 Fig SPIRIT 2013 checklist: Recommended items to address in a clinical trial protocol and other related documents. (PDF)

S2 Fig Interview guides. Semi-structured interview guides used for both participant and clinician interviews.
(PDF)

S3 Fig Participant information and consent forms for study participants and clinician interviews. (PDF)

S1 File (DOCX)

S2 File (PDF)
