# Supplementary material for: Protocol to evaluate a pilot program to upskill clinicians in providing genetic testing for familial melanoma
Source: PLoS One. 2022 Dec 7;17(12):e0275926. doi: 10.1371/journal.pone.0275926 (PMC9728910; doi:10.1371/journal.pone.0275926)
Supplement: S2 Fig — Semi-structured interview guides used for both participant and clinician interviews. (PDF) [file pone.0275926.s002.pdf]

Pre Test Counselling Aides

Genetics 101

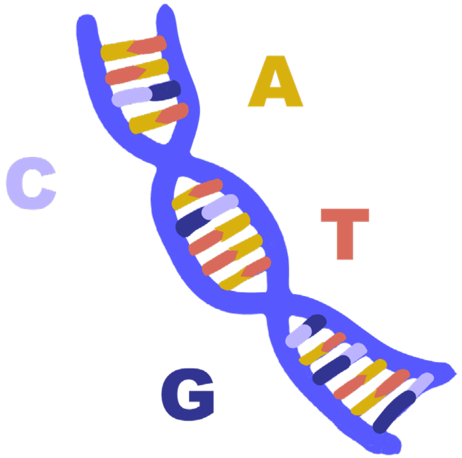

- Everyone has >20,000 genes made up of DNA
- Half come from Mum and half from Dad
- The job of a gene is to code for a protein*
- “Mistakes” in the code affect quantity or quality of protein = Mutations
- Mutations in genes which are important in cell growth can increase our risk of developing cancer

https://creativecommons.org/licenses/by-nc-sa/4.0/

Genetic/Environmental Interactions

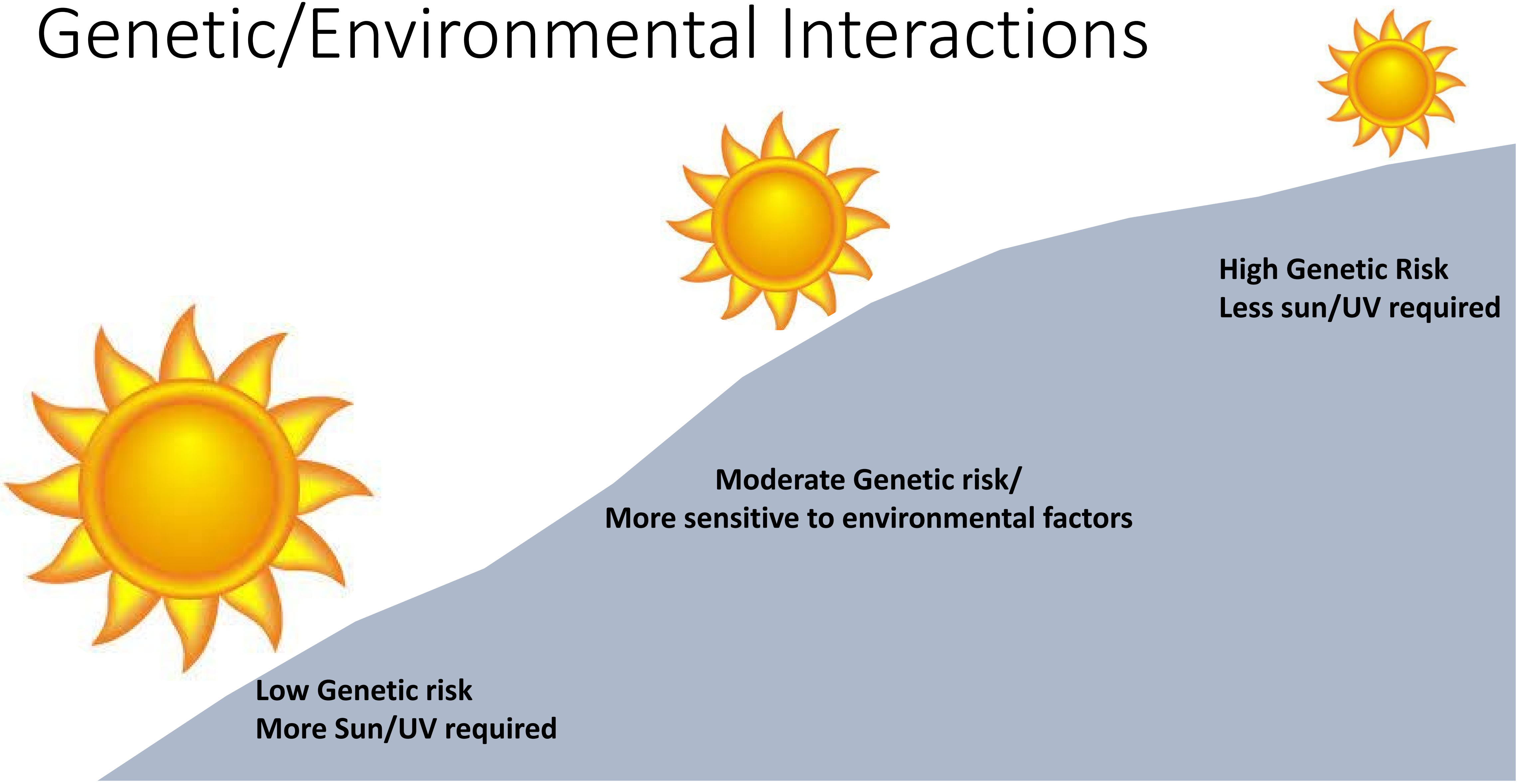

Not everyone who inherits a mutation will get a melanoma or another cancer.
Chance and environmental factors play a role.

https://creativecommons.org/publicdomain/zero/1.0/

“High risk” for inherited melanoma mutations

- Younger ages of diagnosis (<40)
- Single individual with multiple melanomas
- Two or more close relatives who have had melanoma
- A family history of a related cancer
- A family member with more than one type of cancer

High risk genes

- A portion of high risk melanoma families (3+ family members affected) will have a *CDKN2A* mutation
- A smaller percentage will have mutations in other melanoma genes
- Many genes are still unknown

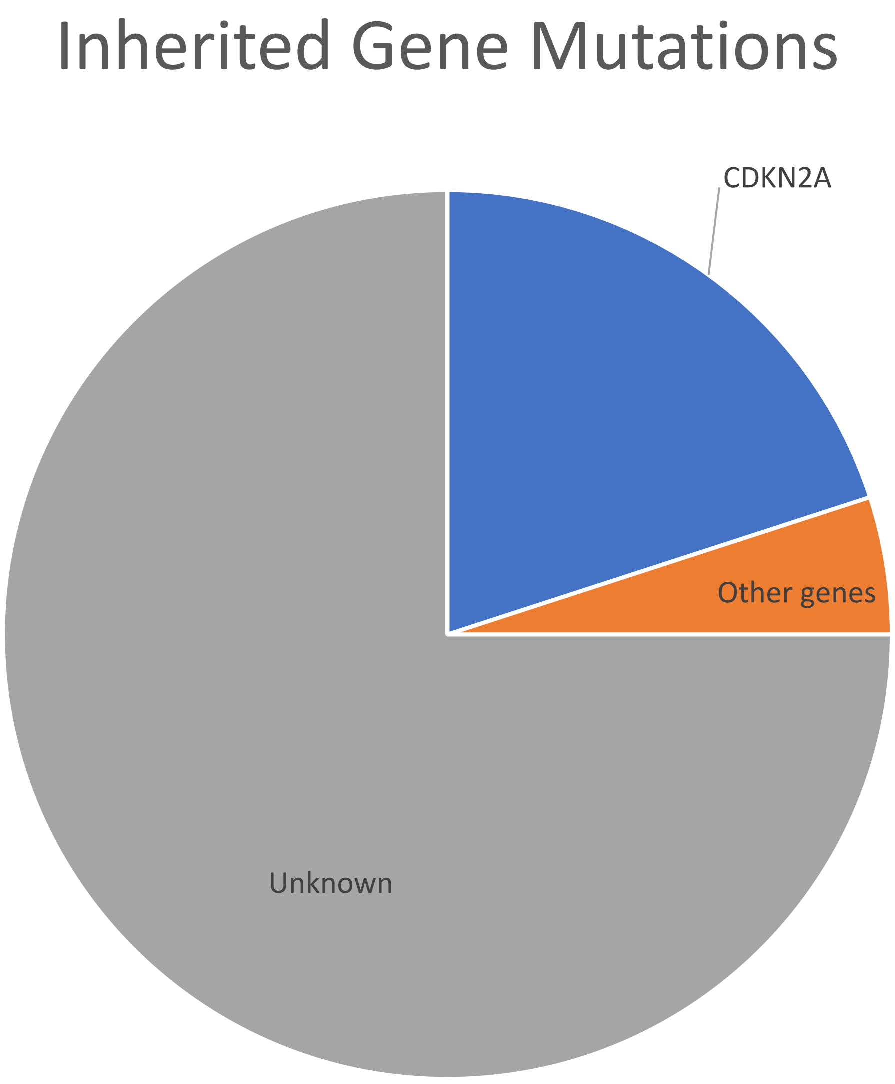

Genetic Testing for Melanoma

- We will be testing over a dozen genes that have been associated with an increased risk for melanoma

- Some of these genes may identify an increased risk for **other** cancers besides melanoma
  - **e.g. we will be testing *BRCA1* and *BRCA2* which are associated with an increased risk for breast and ovarian cancer.**

Approximate Lifetime Cancer Risk

	Risk in the Queensland Population (no family history)	Risks to Known *CDKN2A* Mutation Carriers
Lifetime risk of melanoma (by age of 80 years)	6-8%	50%-70%*
Lifetime risk of pancreatic cancer (by age of 80 years)	1%	<17% - varies between families

* People with variations in the “red hair” gene (*MC1R*) have an increased risk

Possible Results of Genetic Testing

Positive (a definite mutation was found)

- High risk for melanoma and possible other cancers
- Each first degree relative (child, sibling, parent) has a 50% chance of having the same mutation

Negative (no mutation was found)

- Still at increased risk for melanoma based on personal/family history
- There may be a different genetic change causing melanoma in this family
- No genetic testing recommended for your family members at this stage

?

- Variant of Uncertain Significance (a mutation was found but we don't know whether it can cause melanoma)
  - Still at increased risk for melanoma based on personal/family history
  - Additional studies in other family members may be needed to clarify significance

Implications for family members?

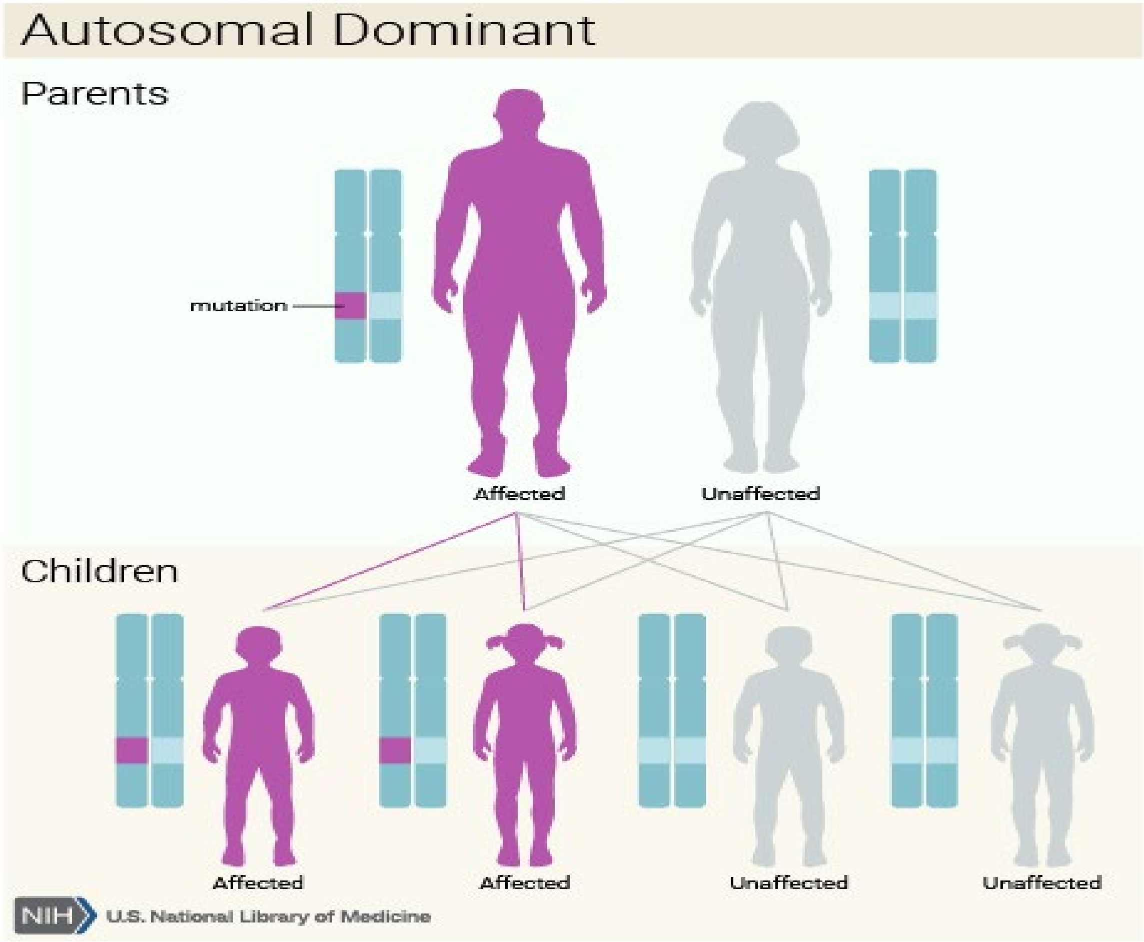

Every 1st degree relative (child, parent, sibling) has an ~50% chance of having the mutation

Credit: U.S. National Library of Medicine

Life Insurance Considerations

- As of July 1, 2019 life insurance companies in Australia **can not** ask an individual to disclose their or their first degree relatives' genetic test results
  - Currently applies to policies that are under $500,000
- If you have a favourable genetic result (You do not carry a mutation that has been identified in your family) this can be used to adjust your premiums
- You do not have to disclose any family health history beyond your 1st degree relatives
- You do have to disclose your personal health history and that of your 1st degree relatives

FSC Standard No. 11 – Moratorium on Genetic Tests in Life Insurance

https://www.fsc.org.au/resources-category/publication/1785-moratorium-key-facts/file

How are you feeling about this information?

Do you have any questions before proceeding with sample collection?

Post-Test Counselling Aides

Genetics 101

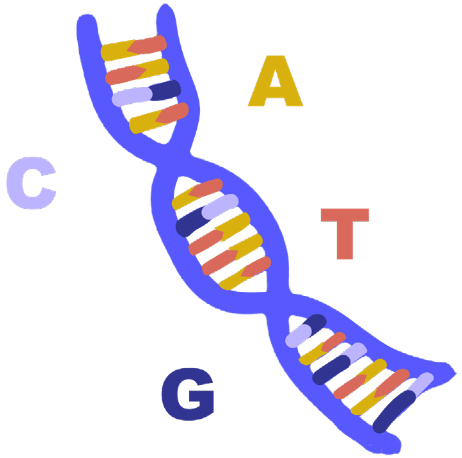

- Everyone has >20,000 genes made up of DNA
- Half come from Mum and half from Dad
- The job of a gene is to code for a protein*
- "Mistakes" in the code affect quantity or quality of protein = Mutations
- Mutations in genes which are important in cell growth can increase our risk of developing cancer

https://creativecommons.org/licenses/by-nc-sa/4.0/

Single mutations in genes

Single base mutation example:

Think of a gene as like a sentence in an instruction manual.

Consider the sentence:

**MUM CUT THE HOT DOG**	The gene works as it should.
MOM CUT THE HOT DOG	Some spelling changes look different but *don't* change the meaning of the sentence. Therefore, the gene still works.
MXM CUT THE HOT DOG	Other spelling changes look different and *do* change the meaning. Therefore, the gene doesn't work.
MEM CUT THE HOT DOG	Other spelling changes look different but we do not know if the meaning changes. Therefore, we are uncertain what the effect on the gene might be.

High risk genes

- A portion of high risk melanoma families (3+ family members affected) will have a *CDKN2A* mutation
- A smaller percentage will have mutations in other melanoma genes
- Many genes are still unknown

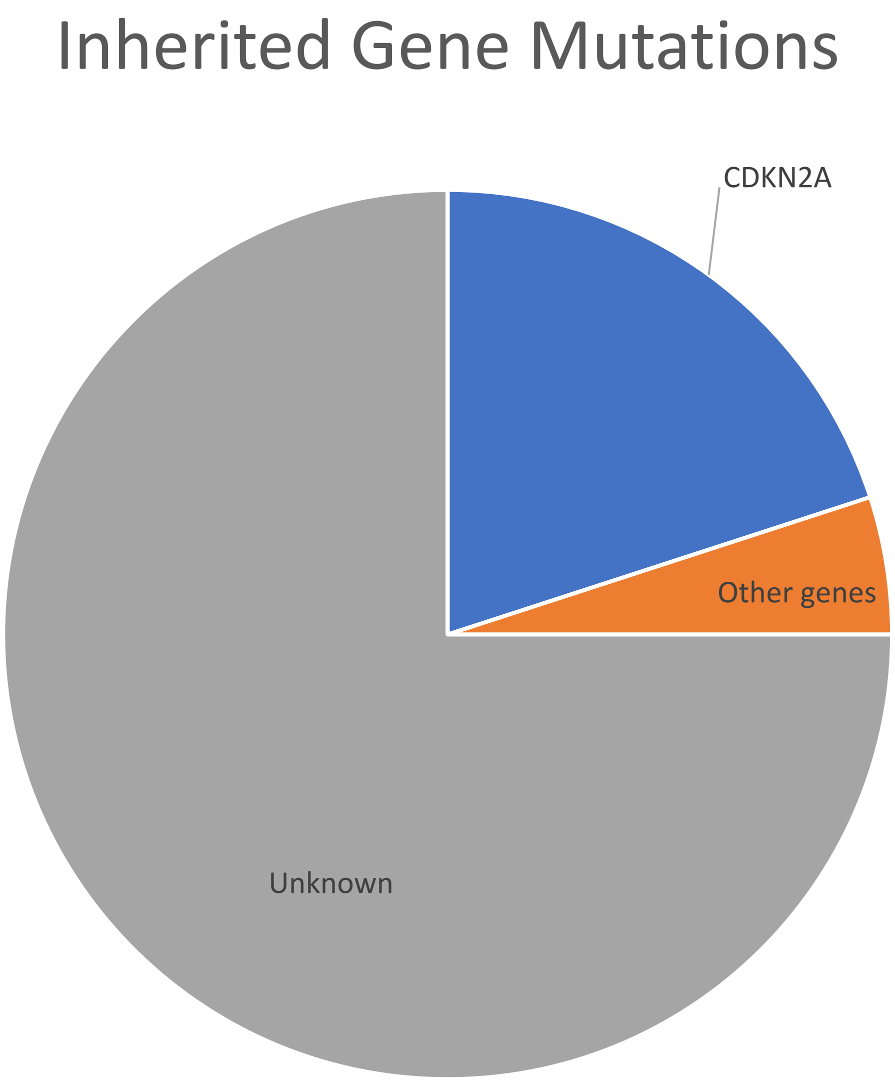

Possible Results of Genetic Testing

Positive (a definite mutation was found)

- High risk for melanoma and possible other cancers
- Each first degree relative (child, sibling, parent) has a 50% chance of having the same mutation

Negative (no mutation was found)

- Still at increased risk for melanoma based on personal/family history
- There may be a different genetic change causing melanoma in this family
- No genetic testing recommended for your family members at this stage

- Variant of Uncertain Significance (a mutation was found but we don’t know whether it can cause melanoma)
  - Still at increased risk for melanoma based on personal/family history
  - Additional studies in other family members may be needed to clarify significance

Is now a good time to receive your results?

Genetic/Environmental Interactions

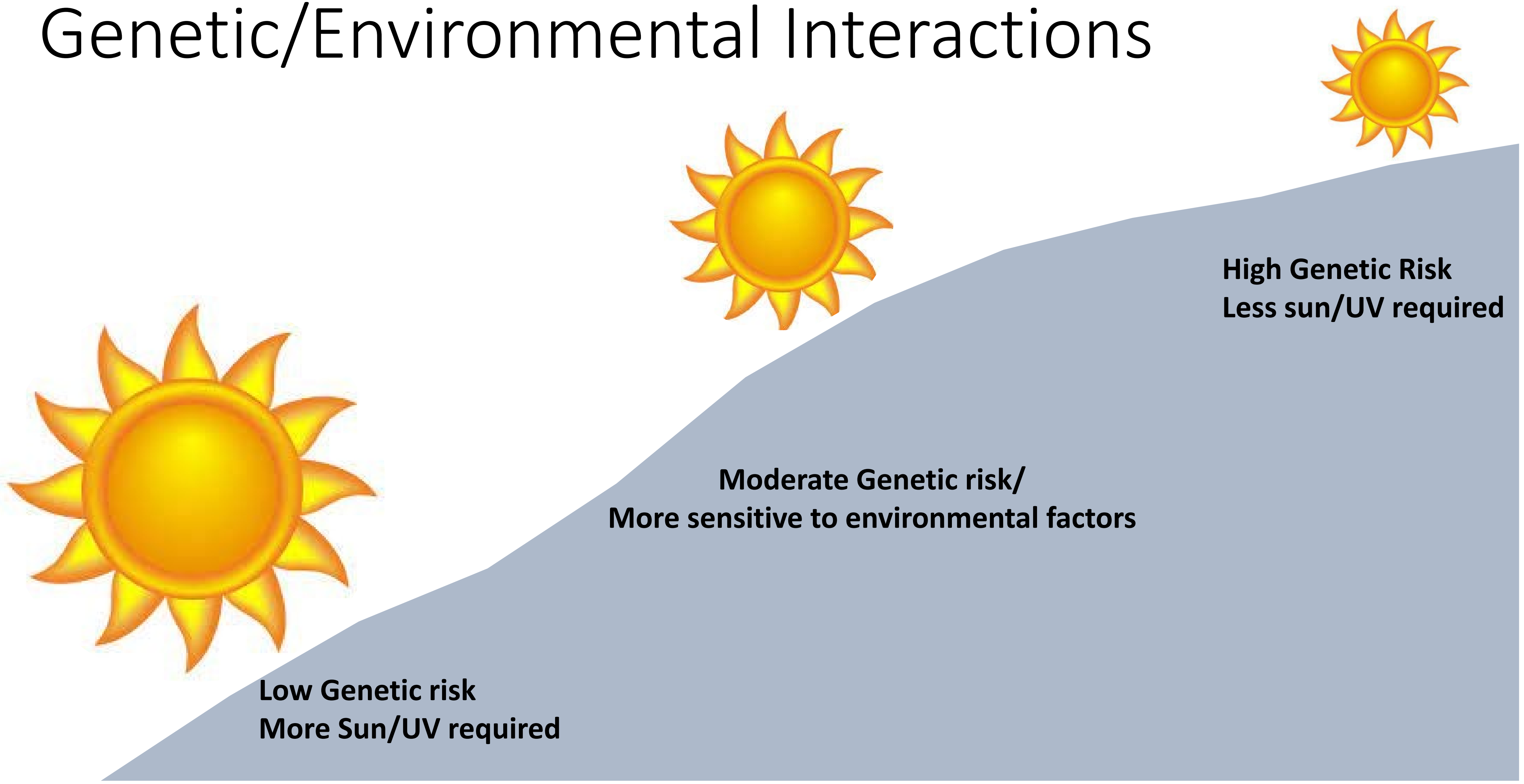

Not everyone who inherits a mutation will get a melanoma or another cancer.
Chance and environmental factors play a role.

https://creativecommons.org/publicdomain/zero/1.0/

Implications for family members?

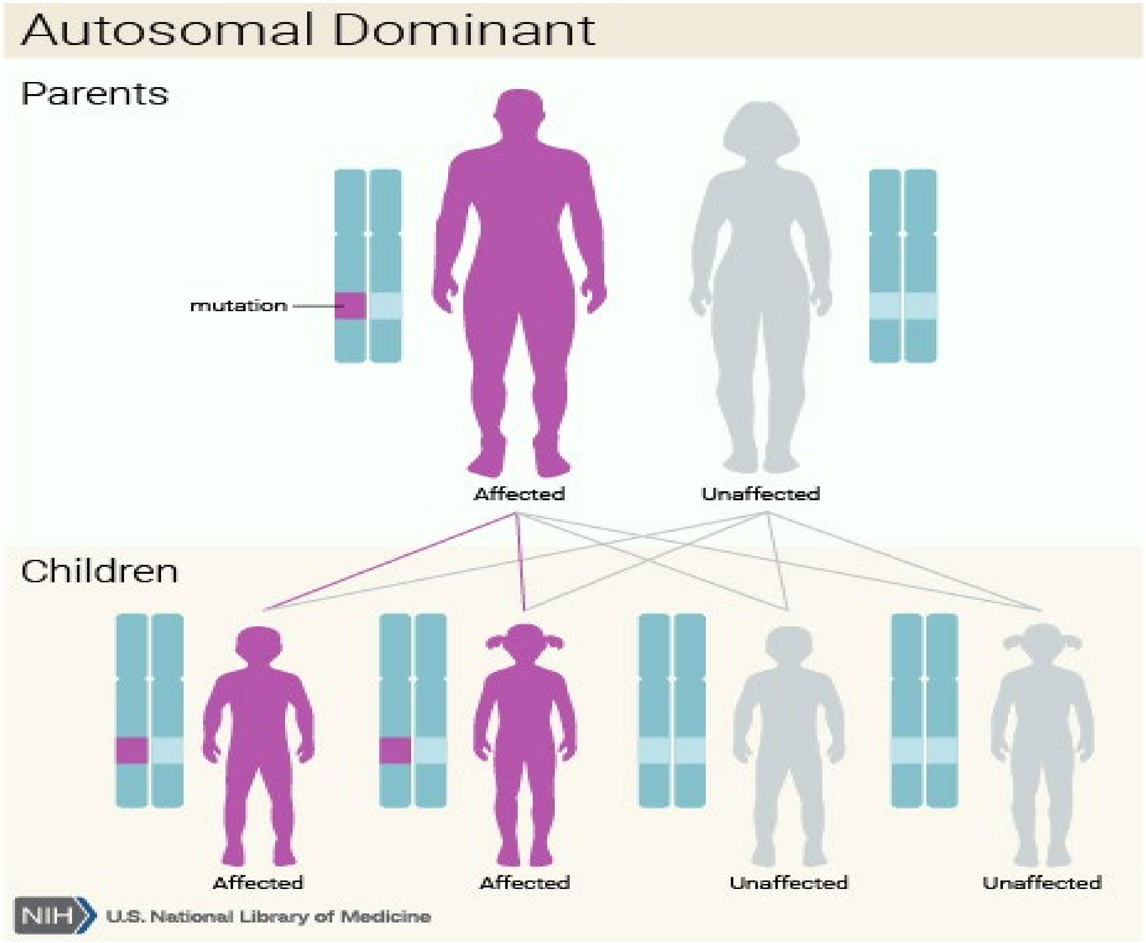

Every 1st degree relative (child, parent, sibling) has an ~50% chance of having the mutation

Credit: U.S. National Library of Medicine

Melanoma Surveillance Recommendations

- All members of families with melanoma should:
  - Regularly check their skin
    - Monthly self- skin exams
    - Total body skin exams with your dermatologist (6 months/annually depending on the history)
  - Take steps to lower UV exposure
    - Wear protective clothing, apply sunscreen, avoid tanning beds and avoid excessive sun exposure
  - Reduce environmental risks
    - No smoking

CDKN2A Approximate Lifetime Cancer Risk

	Risk in the Queensland Population (no family history)	Risk to family history of melanoma and Negative *CDKN2A*	Risk to Known *CDKN2A* Mutation Carriers
Lifetime risk of melanoma (by age of 80 years)	6-8%	~2X the background risk	50%-70%*
Lifetime risk of pancreatic cancer (by age of 80 years)	1%	1%	<17% – varies between families

* People who have variations in the "red hair" gene (*MC1R*) have an increased risk

Pancreatic surveillance for CDKN2A carriers

- *CDKN2A* carriers are at particularly high risk for pancreatic cancer if:
  - They have a positive family history of pancreatic cancer
  - They have a specific mutation associated with familial pancreatic cancer
  - They smoke (past or present)
  - They have sudden, late-onset diabetes
- Screening should include
  - endoscopic ultrasonography(EUS), MRI/magnetic resonance cholangiopancreatography and/or CT (Canto et al 2018)
- Recommendations?
  - Start screening at 55 years or 10 years prior to earliest age of onset in family
  - Frequency unknown. Research studies have performed annually (Canto et al 2018)
  - Do not smoke

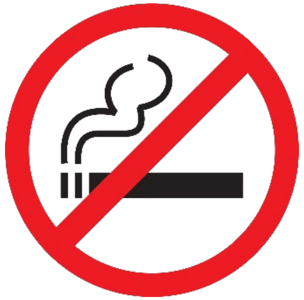

This Photo CC BY-NC

Lucas & Kastrinos 2019 *JAMA* 322 (5) 407 ; Canto et al 2018 *Gastroentererology* 155(3) 740-75

?Additional Surveillance POT1 and POLE

There are no current recommendations because:

1) Recently discovered genes in a small number of families
2) Some people with mutations in these genes have family histories of other cancers
3) We don’t know yet whether this is chance or represent a real risk
4) We also don’t know whether certain, specific mutations in these genes are associated with an increased risk and others are not.
5) We are hoping that research on you, your family and other similar families will help us to understand what additional risks, if any, might need to be monitored.

Life Insurance Considerations

- As of July 1, 2019 life insurance companies in Australia **can not** ask an individual to disclose their or their first degree relatives' genetic test results
  - Currently applies to policies that are under $500,000
- If you have a favourable genetic result (You do not carry a mutation that has been identified in your family) this can be used to adjust your premiums
- You do not have to disclose any family health history beyond your 1st degree relatives
- You do have to disclose your personal health history and that of your 1st degree relatives

FSC Standard No. 11 – Moratorium on Genetic Tests in Life Insurance

https://www.fsc.org.au/resources-category/publication/1785-moratorium-key-facts/file

Review and Questions

- How are you feeling about the information we provided?
- Do you have a good understanding of the information?
- Do the screening recommendations make sense?
- Do you understand the next steps for you/your family?

Standard Operating Procedure (SOP) - Pre and Post counselling for Genetic testing: Dermatologists

**INFORMATION UPDATES (to be performed at both Pre and Post counselling sessions)**			
**Task**	**Details**	**Done**	**Comments**
Personal Contact Update	-Review and update preferred type of contact; update phone numbers, email, mailing/billing address<br>-Review and update the next of kin contact		
Medical History Update	-Review personal medical history including current or recent skin issues and melanoma; other forms of cancer; other medical diagnoses of concern; psychological history<br>-Confirm any new diagnoses with pathology records if possible		
Family History Update/Pedigree Updates	- Review family history of skin cancers (how many per individual, age of diagnosis, location on body; etc.). Correct any previous information; add new information<br>-Confirm any reported diagnoses with pathology records if possible<br>-Obtain details of other types of cancers in 1st and 2nd degree relatives (type of cancer primary; age of diagnoses; treatment types<br>-Known conditions that multiple people in the family have/and formal genetic diagnoses in the family		
**PRE-TEST COUNSELLING**			
**Task**	**Details**	**Done**	**Comments**
Review basic genetics	-DNA- genes – proteins = basic functions of our body<br>-Mutations are "mistakes" in the genetic code that can disrupt protein function<br>-Interplay of genetic (inherited) and environmental factors create cancer<br>-Review how inherited mutations changes can impact the timing of diagnosis, intensity, number of cancers, family history		
Definition of "high risk" families	-Younger age of diagnosis of cancer (<40)<br>-Multiple cancers in a single individual (i.e. multiple primary melanomas/pancreatic cancer and melanoma, etc.)<br>-Two or more close relatives with the same cancers		
Review melanoma genetics	-The knowledge of genetics of melanoma is rapidly expanding but there is still much we do not know.<br>-5-10% of all cases of melanoma are suspected to be familial/inherited<br>-Of those that are familial, ~20% have a mutation identified in the *CDKN2A* gene and others have mutations in other known melanoma susceptibility genes<br>-The background lifetime risk for melanoma in QLD is 6-8%. If there is a known *CDKN2A* mutation, that risk is much higher (~50-70%*)<br>-*Modifier genes can contribute to the risk for melanoma as well. An individual with a *CDKN2A* mutation AND a *MC1R* (red hair gene) mutation has ~70% risk for melanoma.		

Explanation of testing	-We will be using a panel test that investigates genes that have been found to be associated with an increased genetic risk for melanoma.<br>• *CDKN2A, BAP1, BRCA2, CDK4, MITF, POT1, PTEN, RB1, TP53, BRCA1, MC1R, TERT, POLE*<br>-The panel may identify a mutation that increases risks for other cancers/genetic conditions beyond melanoma.<br>-Limitations of testing:<br>• This Invitae panel has high sensitivity and specificity for the coding regions of the genes it examines. It will not pick up all mutations/rearrangements that may influence cancer risk in other (non-coding regions) parts of the gene.<br>• There may be mutations in the genes that are later (through further research) reclassified as pathogenic or likely pathogenic that were not reported.<br>• The panel will not determine all potential modifying mutations that may be contributing to a person's cancer risk.<br>-Not every gene mutation has a known risk for penetrance; therefore some of the information may not provide exact risk modification for cancer or screening options.<br>-Anticipatory guidance about the possible testing results:<br>• Known deleterious mutation identified<br>• Mutation identified that we do not understand the implications of (variant of uncertain significance)<br>• No mutation identified		
Implications for family members	-Mutations are inherited in an autosomal dominant pattern. If the participant is identified to have a mutation, there is a 50% each of their first-degree relatives also has it.<br>-Having the mutation does not necessarily mean the person will get cancer, there will be a modified lifetime risk for cancer.<br>-Family members will need to make their own decisions about testing but it would be suggested that the participant considers sharing their genetic testing information with family members.		
Psychosocial implications	- Participant may feel different emotions regarding heritability of cancer and potential genetic results. It is best to have a conversation with the participant before testing so they can better anticipate how they may feel with various results.<br>- Check in with participant and how they feel about the reason they have cancer… what is their self-belief system?<br>-Determine support system and coping mechanisms<br>• Who will they discuss their decision about testing with?<br>• Who will they discuss their testing results with?<br>• How do they cope with medically stressful/uncertain situations?		

	-Provide some anticipatory guidance regarding some emotions peoples may feel when faced with various genetic test results:<br>• Anger at self for waiting so long to seek an underlying cause;<br>• Relief at having an answer for "why me?";<br>• Guilt for potentially passing on potential genetic mutation;<br>• Disappointment when there is not an answer through genetic testing;<br>• Etc.		
Insurance Considerations	-Review the information from the FSC Standard 11 – Moratorium on Genetic Tests in Life Insurance<br>- Policies up to 500K cannot request information on genetic test results in the individual or their first-degree relatives<br>- A favourable result in a family member can be used to reduce premiums<br>- A participant must still disclose personal and family health conditions (e.g. melanoma diagnosis)<br>- Policies over the 500K can ask for genetic testing results<br>- This has not been tested in any court proceedings to date<br>- No government oversight<br>https://www.fsc.org.au/resources-category/publication/1785-moratorium-key-facts/file		
Establish the Plan for Resulting	-The study coordinator will discuss the plan with participant for receiving the results - return appointment booked for results session<br>-Review expected time-frame for results (~4 weeks)<br>-Discuss if other family members may want to attend the result session<br>-Establish best form of contact and request a next of kin contact especially for participants who are terminal		
Obtain sample/provide testing request	-The study coordinator will assist in completion of paperwork, sample collection and sample shipment.<br>-PLEASE NOTE: INVITAE CAN'T ACCEPT SAMPLES FROM PARTICIPANTS WHO HAVE HAD ALLOGENIC BONE MARROW/STEM CELL TRANSPLANTS<br>-PLEASE NOTE: PARTICIPANTS WHO HAVE AN ACTIVE HAEMATOLOGICAL MALIGNANCY (e.g. Leukemia), OR A RECENT BLOOD TRANSFUSION MAY NOT BE ELIGIBLE FOR COLLECTION		
**POST-TEST COUNSELLING – Known deleterious mutation (Positive Result of a Pathogenic or Likely Pathogenic Mutation)**			
**Task**	**Details**	**Done**	**Comments**
Schedule results session and prepare for visit	-The study coordinator will confirm the results session and remind participant they can bring support family members/people<br>-The clinician will review the results from the laboratory and discuss details with the study genetic counsellor regarding the specific result.<br>-the study coordinator/genetic counsellor will prepare a written document for follow-up screening based on results that will be discussed with participant and taken home for future reference		

Explanation of Test results	-A mutation that is known to be associated with increased cancer risk has been identified.<br>-Review the mutation that has been identified and the gene in which it was found<br>-Based on the particular gene involved, the relative risk for melanoma in a person's lifetime will be different – refer to the written information from the laboratory/study genetic counsellor for more information<br>-Based on the gene and mutation involved, there may be an increased risk for other cancers/genetic conditions (i.e. mutations in CDKN2A can incur an increased risk for pancreatic cancer but that risk may vary based on the specific mutation identified and the participant's family history)		
Implications for family members	-Family members can now have targeted, informative testing if they choose to<br>• If they do not have the mutation, they are still at increased risk for developing melanoma but it is not as high as those who carry the mutation.<br>• If they have the mutation they can start screening appropriately for the conditions they are at increased risk for<br>- Discuss cascade testing as needed<br>• Identify the at-risk relatives that are at 50%<br>• Explain the rationale for testing 50% risk first (i.e. children rather than grandchildren)<br>-Review screening recommendations for family members based on the type of result found and family history<br>-Offer to provide written information regarding the mutation identified in the family for the participant to explain the finding to family members<br>-Offer referral for genetic counselling/geneticist review for family members		
Screening recommendations	-Review with the participant although a genetic implication has been identified, we know that cancer has significant environmental interplay. Therefore, preventative measures through sun safety and screening are still paramount for reducing the risks of cancer.<br>-Explain any screening that is now recommended based on the potential increased risk for other cancers (e.g. breast, bowel, pancreatic cancers)<br>-Review current melanoma screening protocol<br>-Discuss any currently available research for screening options available for people at high risk for melanoma		
Psychosocial Implications	Ask the participant how they feel with the result they have been given<br>• Every participant will react to results in a different way depending on their personal experiences and preconceived ideas about testing.<br>• Many participants have an idea of what their results will likely be, sometimes the reality can be difficult to acknowledge<br>-Explore how this result will impact their feelings towards themselves; their family; their medical health; their screening and preventative actions.		

	-Assess the participant for any psychological issues regarding accepting the results – provide referrals to mental health specialists as required.		
**POST-TEST COUNSELLING – No mutation identified (Negative Result)**			
**Task**	**Details**	**Done**	**Comments**
Schedule results session and prepare for visit	-The study coordinator will confirm the results session and remind participant they can bring support family members/people<br>-The clinician will review the results from the laboratory and discuss details with the study genetic counsellor regarding the specific result.<br>-the study coordinator/genetic counsellor will prepare a written document for follow-up screening based on results that will be discussed with participant and taken home for future reference		
Explanation of Test results	-No mutation was identified in the genes that were tested.<br>-This does not eliminate the risk of a familial mutation in other genes or within the genes beyond the level of testing that was performed.<br>-Participants should maintain contact with genetic services and be offered additional testing in the future as the technology advances.		
Implications for family members	-Review screening recommendations for family members based on the family history<br>-Negative results need to be shared with family members so they understand they do not need to be tested.<br>• In some scenarios it may be warranted to offer testing (or more detailed sequencing) to other family members who have a more significant history of multiple melanoma and or pancreatic cancer to reduce the risk of the proband being a phenocopy in a high risk family. Review the family history with the study genetic counsellor to determine if this is appropriate in your participant's family.		
Screening recommendations	- Review the screening recommendations for the participant based on their personal and family history of melanoma/other cancers.<br>-Negative results do not mean screening is no longer needed, particularly for melanoma or a cancer which is known in the family.		
Psychosocial Implications	- Ask the participant how they feel with the result they have been given<br>• Every participant will react to results in a different way depending on their personal experiences and preconceived ideas about testing.<br>• Many participants have an idea of what their results will likely be, sometimes the reality can be difficult to acknowledge<br>-Explore how this result will impact their feelings towards themselves; their family; their medical health; their screening and preventative actions.<br>-Assess the participant for any psychological issues regarding accepting the results – provide referrals to mental health specialists as required.		

POST-TEST COUNSELLING – Variant of Uncertain Significance – NOT LIKELY IN THIS STUDY BASED ON PANEL REQUIREMENTS			
**Task**	**Details**	**Done**	**Comments**
Schedule results session and prepare for visit	-The study coordinator will confirm the results session and remind participant they can bring support family members/people<br>-The clinician will review the results from the laboratory and discuss details with the study genetic counsellor regarding the specific result.<br>-the study coordinator/genetic counsellor will prepare a written document for follow-up screening based on results that will be discussed with participant and taken home for future reference		
Explanation of Test results	-A mutation has been found but it has an unknown effect on gene function.<br>-Carefully review the explanation of the classification of the variant provided by the testing laboratory/the study genetic counsellor.<br>-Explore any additional research studies that may help clarify the findings.<br>-This does not eliminate the risk of a familial mutation in other genes or within the genes beyond the level of testing that was performed.		
Implications for family members	-Genetic testing results of VOUS should be shared with family members so they can be aware of the testing that has occurred. In most instances there is no reason to test family members for this mutation. However, family studies may make this finding more informative if there are a number of affected relatives – contact the study genetic counsellor to inquire about potential research studies.<br>-Review screening recommendations for family members based on the family history		
Screening recommendations	- Review the screening recommendations for the participant based on their personal and family history of melanoma/other cancers.		
Psychosocial implications	-Ask the participant how they feel with the result they have been given<br>• Every participant will react to results in a different way depending on their personal experiences and preconceived ideas about testing.<br>• Many participants have an idea of what their results will likely be, sometimes the reality can be difficult to acknowledge<br>-Explore how this result will impact their feelings towards themselves; their family; their medical health; their screening and preventative actions.<br>-Assess the participant for any psychological issues regarding accepting the results – provide referrals to mental health specialists as required.		
