# Supplementary material for: Protocol to evaluate a pilot program to upskill clinicians in providing genetic testing for familial melanoma
Source: PLoS One. 2022 Dec 7;17(12):e0275926. doi: 10.1371/journal.pone.0275926 (PMC9728910; doi:10.1371/journal.pone.0275926)
Supplement: S3 Fig — (PDF) [file pone.0275926.s003.pdf]

<u>Semi-structured Interview Guide</u>

Study title: Investigation into the psychosocial and behavioural impact of genetic testing for familial melanoma.

<u>Introductory script</u>

- Thank participant for agreeing to take part in this study
- Remind the participant that the purpose of this interview is to discuss their experiences in the study so far, and experience with familial melanoma
- Discuss the role of the interviewer: to raise topics for discussion and then to listen as the participant shares their views and experiences.
- Reassure the participant that they are free to talk about any aspect of their experience or attitudes. There are no right or wrong or even typical answers to any of the questions that we will discuss.
- Remind the participant that, with their permission, the interview will be audio-taped.
- Reassure confidentiality and the participant's right to stop the interview at any time.
- Clarify that the interview will take approximately 30 minutes to 1 hour.
- Ask whether the participant has any questions before we begin.

Melanoma in the family

1. Can you tell me about how melanoma has affected your life?
2. Can you tell me about when you first became aware that melanoma was 'in your family'? Do you recall it having any impact on your childhood? In what way?
3. How is melanoma discussed amongst your family members?

Decision to have genetic testing

1. Can you tell me about your decision regarding whether to accept or decline genetic testing for this study?
2. What types of conversations did you have with your family members about this study and genetic testing?
3. In your opinion, what are the benefits of having genetic testing for melanoma risk?
4. In your opinion, what are the drawbacks of having genetic testing for melanoma risk?

The testing process

1. How useful was the pre-test counselling session?
2. The genetic test results indicated you have a (e.g. CDKN2A) mutation. What is your understanding of what that result?
3. How did hearing this result make you feel?
4. Has receiving these genetic test results had any impact on your everyday life or behaviour? If Yes, in what way?

5. How much do you feel that genetic testing added to this study? Would the education and counselling sessions alone achieved the same outcome for you?
6. Could you please tell me a little about your experience of receiving genetic counselling over video-conferencing?
   Prompt questions: “Do you think video conferencing is acceptable as an alternative to face-to-face consultation?”,
   “How easy was it for you to use the video-conferencing?”,
   “How satisfied were you with video-conferencing?”
7. Would you recommend this study to others? Why?

Psychological impact / genetic fatalism

1. How has your participation in this study affected your beliefs about your own personal risk of melanoma?
2. How much of a role do you think that chance or luck plays in your melanoma risk?
3. Do you feel that sun-protective behaviours can modify melanoma risk in people who carry a genetic mutation?
4. Do you believe that skin checks affect melanoma risk for people who carry a genetic mutation?
5. In general, do you believe that it is possible for you to prevent future melanomas? Why or why not?
6. Do you feel that a person who carries a mutation is destined to get melanoma regardless of their health behaviour?

Interview debriefing and closure script

- ‘I have no further questions. Is there anything else you would like to bring up, or ask about, before we finish the interview’
- Thank the participant for sharing their experiences/
- As how the participant felt taking part in the interview.
- Do they still have the telephone number in case they would like to contact us in the future?
- Reminder of the future questionnaires (3 months and 12 months) that will be emailed to them as part of this study.

Participant Name: Participant ID: Date/time:

Semi-structured interview guide for clinicians

Study title: Investigation into the psychosocial and behavioural impact of genetic testing for familial melanoma.

Introductory script

- Thank participant for agreeing to take part in this study.
- Introduce the study and briefly discuss its purpose.
- Discuss the role of the interviewer: to raise topics for discussion and then to listen as the participant shares their views and experiences.
- Reassure the participant that they are free to talk about any aspect of their experience or attitudes. There are no right or wrong or even typical answers to any of the questions that we will discuss.
- Remind the participant that, with their permission, the interview will be audio-taped.
- Reassure confidentiality and the participant's right to stop the interview at any time.
- Clarify that the interview will take approximately 30 minutes.
- Ask whether the participant has any questions before we begin.

Topic: training process

"You'll remember that our training process involved an initial presentation of pre-test genetic education and counselling, which included a mock counselling appointment; then you observed a genetic counsellor lead a real appointment, and then a genetic counsellor observed you leading an appointment and provided debriefing after. And a similar training process was used at a later date for returning genetic test results."

- Could you tell me your thoughts on the training process?
- What aspects of the training process were most useful?
- What aspects of the training process were least useful?
- How well prepared did you feel by the end of the training?
- was there anything missing from training process?
- In hindsight, was there aspect of the training which you think was unnecessary?
- How useful were the counselling aides?
- Could they be improved?
- How useful were the Pre and Post Test Counselling SOPs (standard operating procedures)?
- Do you think that receiving this training will impact you in the long-term? If so, how?
- How likely would you be to recommend this training or similar training to other clinicians? Why?

Topic: genetic testing for familial melanoma

- What is your opinion on the usefulness of providing genetic testing for familial melanoma?
- What is your opinion on the limitations of providing genetic testing for familial melanoma?
- Are there aspects of providing genetic testing for familial melanoma that concern you?
- How do you feel about familial melanoma genetic testing being offered in dermatology clinics?
- Based on your experience in the field so far, what would be the challenges to implementing genetic testing in dermatology clinics?
- What advantages are there to offering familial melanoma genetic testing in dermatologists clinics?
