# Supplementary material for: Protocol to evaluate a pilot program to upskill clinicians in providing genetic testing for familial melanoma
Source: PLoS One. 2022 Dec 7;17(12):e0275926. doi: 10.1371/journal.pone.0275926 (PMC9728910; doi:10.1371/journal.pone.0275926)
Supplement: S1 File — (DOCX) [file pone.0275926.s004.docx]

**Clinical Study Protocol**

**Study title:** Investigation into the psychosocial and behavioural impact of genetic testing for familial melanoma

**Principal Investigator**

Dr Aideen McInerney-Leo^1^

**Associated Investigators**

Clare Primiero^1^

Betsy Peach^1^

Dr Tatiane Yanes^1^

Prof H. Peter Soyer^1^

Prof Nick Hayward^2^

Jane Palmer^2^

**Investigator Affiliation**

1. Dermatology Research Centre, The University of Queensland, Diamantina Research Institute, Brisbane, Australia
2. QIMR Berghofer Medical Research Institute, Brisbane, Australia

**Protocol Author:** Clare Primiero

**Study site:** The Clinical Research Facility, at Princess Alexandra Hospital.

**Protocol Code Number:** AML001

**Date:** 12/08/2021

**Version:** 4

**Table of Contents**

[1. Introduction 4](#_Toc35332509)

[**1.2 Aims** 5](#_Toc35332510)

[**2.** **Study Design** 6](#_Toc35332511)

[**2.1 Methods** 6](#_Toc35332512)

[**2.2 Recruitment** 6](#_Toc35332513)

[**2.3. Study Visits** 8](#_Toc35332514)

[**3.** **Genetic Counselling conducted by video conferencing** 11](#_Toc35332515)

[**4.** **Participants who decline genetic testing** 11](#_Toc35332516)

[**5.** **Dermatologist reporting genetic results** 11](#_Toc35332517)

[**6.** **Data Linkage** 12](#_Toc35332518)

[**7.** **Genetic Sequencing** 12](#_Toc35332519)

[**8.** **Data Analysis** 12](#_Toc35332520)

[**9.** **Data Management** 13](#_Toc35332521)

[**10.** **Dissemination of results** 13](#_Toc35332522)

[**11.** **Ethical considerations** 14](#_Toc35332523)

[**12.** **References** 15](#_Toc35332524)

# Introduction

Recent advances in genetic sequencing technology have made the process more affordable and quicker, making the technology more accessible for use in healthcare. Furthermore, online Direct-to-Consumer (DTC) genetic testing is becoming increasingly more popular and is marketed under the pretext of ‘recreational’ purposes. With interest and demand for genetic testing increasing, and as the field of precision medicine is steadily growing, the investigation of the psychosocial consequences of genetic testing is critical. Currently, genetic testing is routinely used for known single gene, autosomal dominant disorders such as Huntington’s disease. While procedures for testing for colorectal, breast and ovarian cancer susceptibility mutations has become common in clinical practice, few other cancers have similar processess. The primary objectives of predictive genetic testing for cancer susceptibility is to inform an individual of their increased risk, offer protective or risk-reducing interventions, and where available, appropriate surveillance. For specific cancers where environmental and/or behavioural factors affect risk e.g. lung cancer and skin cancers, testing has the potential to motivate people to change health behaviours to reduce their risk, or delay the onset of disease. A primary concern of genetic testing for cancer susceptibility is the possible negative psychosocial impact of reporting unfavourable results. Before implementing any predictive genetic testing protocols in healthcare, there is an obligation to ensure feasibility and that the benefits outweigh potential harms.

Melanoma is Australia’s 4^th^ most common cancer, with over 13,000 new cases diagnosed in 2016, and over 1,700 deaths annually (AIHW)^1^. Approximately 10% of primary melanoma patients have at least one (and usually only one) first degree relative with a previous melanoma diagnosis^2^. Familial melanoma is often described as having at least two first-degree (FDR) or second degree (SDR) relatives with a melanoma diagnosis. However in Australia, where incidence of sporadic melanoma are highest, familial melanoma is more often defined as having three or more relatives with melanoma or pancreatic cancer within one side of a family^3, 4^. Worldwide, it is estimated that 2-5% of melanoma cases would be attributed to a true inherited familial risk resulting from a high penetrance germline mutation^5, 6^. Between 20 to 40% of these familial melanoma cases are found to be associated with a *CDKN2A* mutation^4^. Further high penetrance genes that have been identified, including *CDK4, MITF, BAP1, TERT, POT1, ACD, TERF2IP, POLE* are rare, each associated with <1% of familial melanoma cases^7^. It is expected that a subset of familial melanoma cases would be attributed to multiple low penetrance risk variants (polygenic inheritance), and/or shared environment^5, 8^.

In Australia, the majority of genetic testing for familial melanoma has been conducted in a research setting. Presently, in most cases the inclusion of genetic testing does not impact clinical management, as someone with extensive personal history would be managed as ‘high risk’ regardless of identified germline mutation. Therefore, the clinical utility of genetic testing for familial melanoma has been questioned^9^. Others have highlighted the benefit of genetic testing for ensuring appropriate surveillance in as yet, unaffected family members^10^. Clinical research studies, however, have continued to show positive outcomes for predictive testing in familial melanoma. Clinical trials have reported that any negative psychological impact of genetic testing is short term^10^, and learning of a genetic susceptibility has provided motivation for greater sun protective behaviour and screening^11^. Furthermore, reporting of negative *CDKN2A* mutation status provides relief for members of *CDKN2A* families, and assists them in re-adjusting to appropriate prevention strategies if previously overly cautious^12^. Importantly, there has been no evidence to suggest that a negative result has instilled a false sense of security, with several studies showing sun protective behaviour and skin examinations did not decrease^10, 13^, and in some studies, actually increased after disclosure of negative mutation status^14, 15^.

Currently, it is recommended that pre and post genetic education and counselling accompanies test reporting, and is provided by a qualified genetic counsellor. However, considering clinical genetic testing is very likely to increase quickly in the near future, it is important to acknowledge logistical implications and consider alternatives for clinical implementation. Counselling and reporting of genetic test results for the *BRCA1*/2 gene by trained nurses or doctors has become routine practice in several oncology clinics in Australia and the UK^16, 17^. Feedback from patients and nurses has been positive and this model provides improved access to genetic testing^16^. Testing and reporting on the *CDKN2A* gene variant is a candidate for mainstreaming the procedure in dermatology clinics. The advantages that a genetic counsellor has in delivering genetic test results is their expertise in dealing with complex issues of inherited risk, unexpected or incidental findings and inconclusive results such as ‘variants of unknown significance’. The mode of inheritance and risk significance is well understood for *CDKN2A*. Furthermore, a positive CDKN2A result would not be unexpected as a strong personal and family history of melanoma is a prerequisite for testing. With appropriate training on providing genetic education and counselling, testing for *CDKN2A* could become a standard procedure for a dermatologist. In most cases, a dermatologist will already know their patient’s personal and family history and are in the best position to offer subsequent recommendations based on test results, such as skin examination intervals. The present study will include a pilot study to produce preliminary data to explore this concept.

Some aspects of reporting personal genetic results for familial melanoma remain to be investigated, or completely understood. Psychosocial aspects such as genetic fatalism or essentialism for this cohort has not been reported. Genetic fatalism or essentialism can be described as the belief that one has little control over health outcomes, and that personal future health is determined by their genetic code^18^. These beliefs are often associated with decreased motivation for protective health behaviour^19^. Furthermore, it is useful to identify psychological traits present in this cohort that are associated with the uptake of genetic testing, and any correlation with significant psychological impact or behaviour change. Additionally, it is unclear what impact genetic testing has in familial cases where a clear line of inherited risk is present, however no responsible mutation is identified, leaving the cause or level of personal risk unexplained. In the present study, the effect of genetic education, counselling and testing, and on genetic fatalism will be explored. Furthermore, the extent to which psychological and behavioural outcomes vary depending on whether there is an identified familial mutation will also be explored. Lastly, this study will evaluate whether the method of delivery (dermatologist compared with genetic counsellor) of genetic test results for familial melanoma influences (or is associated with) psychological and behavioural outcomes.

# **1.2 Aims**

Primary Aim: To understand and describe the participant’s experience with genetic education, counselling and testing, and living with familial melanoma through qualitative analysis of semi-structured interviews.

Secondary Aims: To identify psychosocial and behavioural impact of genetic testing in familial melanoma, using quantitative analysis of validated questionnaires guided by the Health Belief Model as a theoretical framework. This will be achieved by;

1. Evaluate psychosocial and behavioural impact of genetic education, counselling (and testing) in individuals with a familial melanoma, regardless of whether a causal mutation has been identified.
2. Explore genetic fatalism in a subset of participants and whether they feel that genetic counselling (+/- testing) affected their beliefs.
3. Determine whether the person conducting the counselling session (genetic counsellor/dermatologist) affects psychosocial and behavioural outcomes.
4. Evaluate what psychological traits are associated with uptake of testing, psychological impact and behavioural change.
5. Assessment of the clinician training protocol, and supporting documents, for providing genetic testing.

# **Study Design**

# **2.1 Methods**

This study will be completed in two stages with two separate cohorts.

Stage 1: Genetic testing and counselling, semi-structured interview, and questionnaires at multiple time points. (n=50)

Stage 2: A one-off questionnaire designed after analysis from stage 1. (n=200)

The cohort for stage 1 of the study will be made up of approximately 50 participants (depending on when data saturation is reached in qualitative analysis of interviews). These participants will all have a suspected inherited risk of melanoma, and will be further stratified into 3 groups for data analysis, including a) those with a positive CDKN2A mutation, b) those positive for a different mutation for melanoma risk, and c) those where no mutation is found. All groups will receive pre and post genetic education and counselling, questionnaires at multiple time points (see table 2), and will participate in a semi-structured phone interview to discuss their experience with genetic testing and counselling one month after receiving test results.

Interview data will be transcribed and evaluated qualitatively using thematic analysis. Questionnaires will compose of validated instruments (see table 1), and will be analysed as per each scale’s manual and standard procedures. Findings produced by questionnaire and interview analysis will then be used to design a novel questionnaire instrument for capturing psychosocial and behavioural impact of genetic testing has for familial melanoma.

Stage 2 of the study will validate the novel questionnaire instrument in a cross-sectional study, enrolling a second cohort of approximately 200 participants with suspected familial melanoma. This second stage of the study will provide a more complete dataset from which hypotheses and conclusions about the psychosocial and behavioural impact genetic testing has for familial melanoma. This information will be a valuable addition to the current literature and inform genetic testing practices in the future.

# **2.2 Recruitment**

*2.2.1: Stage 1: Genetic counselling and education, questionnaires and phone interview*

Eligibility Criteria: Individuals who are identified as having a strong personal and/or family history of melanoma indicative of inherited risk, will be invited to join the study. The eligibility criteria is based on a recent publication by Taylor *et al* (2019), which describes a tool GenoMELPREDICT that can be used to predict *CDKN2A* mutational status in people from melanoma-prone families^20^. The criteria for a populations with moderate-to-high incidence of melanoma is 3 primary melanomas in an individual and/or families with ≥1 invasive melanoma and ≥2 other invasive melanoma and/or pancreatic cancer among first-degree or second-degree relatives on the same side of the family.

Individuals will be recruited from four sources;

1. A registry of previous participants kept by University of Queensland’s Dermatology Research Centre (UQ-DRC)
2. Selected QIMR Berghofer Medical Research Institute (QIMR Berghofer)’s  *Pathways from Genotype and Environment to MELANOMA*  study (ERM Project ID 25706 HREC/14/QPAH/495)
3. Family members of enrolled participants (with participants’ prior approval)
4. Referrals from local dermatologists or healthcare providers

Previous study participants of the UQ-DRC who are eligible for the study, will be contacted by phone and given a description of the current study to gauge interest in participation. A participant information and consent form (PICF) will be emailed out if there is interest in participating. Individuals will be encouraged to read through the document and discuss with family members and/or healthcare providers. Individuals will be recontacted 1-2 weeks after PICF has been sent out to discuss participation in the study, and to answer any questions individuals have. A time and date will be made for an appointment at the Clinical Research Facility at PA Hospital, at the participant’s convenience.

Researchers at QIMR Berghofer have conducted a study into the genetics associated with melanoma risk. The QIMR Berghofer study does not include reporting genetic results back to their participants. However the research team would offer selected participants who are interested in genetic testing who meet the study testing criteria. The study coordinators at QIMR Berghofer will be supplied with a recruitment brochure to distribute. The brochure will provide contact details for our study team, and will instruct the reader to either call or email our team if they are interested in participating. Upon contact from a QIMR Berghofer study participant, we will then follow the above procedure used for previous UQ-DRC participants for recruitment.

Family members of enrolled participants, will be invited to join the study through the proband (individual who first enrolled). The proband will be asked at the baseline visit if they would like to invite any family members. During the proband’s baseline visit, a genetic counsellor will identify other family members that meet testing criteria, based on a pedigree of family history of cancer. We will provide participants with brochures to give out to interested family members. Upon contact from family members, the same procedure or recruitment will be followed, e.g. phone conversation, emailed PICF, follow-up call to answer questions and book in an appointment.

Doctor referrals for people who are high risk of melanoma, and are interested in participating in research are regularly received at the UQ-DRC. Therefore we would like to have an option of recruiting eligible individuals referred to our research group in this way. In these instances, the individual would be contacted by phone and given a description of the current study to gauge interest and determine eligibility. Thereafter the same procedure for recruiting previous study participants will be followed, as detailed above.

*2.2.2: Stage 2: Cross-sectional newly developed questionnaire*

Eligibility criteria: Individuals who are identified as having a strong personal and/or family history that would meet the criteria for genetic testing as per the GenoMELPREDICT tool^20^.

Individuals will be recruited from the following sources;

1. A registry of previous participants kept by University of Queensland’s Dermatology Research Centre (UQ-DRC), who did not participate in Stage 1 of this study.
2. Registries kept by Australian research collaborators, of individuals who would fit the above inclusion criteria.

An email will be sent to eligible individuals, which will include a description of the study objectives and time involvement required to complete the questionnaire. The email will provide a web-link which will direct the individual to a website that incorporates the informed consent process and the questionnaire, to be completed online at the participant’s convenience.

*The questionnaire and the consenting process, and the research collaborators that will facilitate recruitment, and any other related documents for Stage 2, will be submitted to the HREC committee as an amendment after Stage 1 of the study is complete. This delay is necessary as the questionnaire development is based on outcome analysis of the first stage of the study.*

# **2.3. Study Visits**

*2.3.1. Genetic education and counselling, questionnaires and phone interview (Stage 1, Cohort 1)*

Genetic education and counselling occurs over 2 study visits at the Clinical Research Facility, based at Princess Alexandra Hospital (CRF-PAH), or alternatively over 2 online video conference sessions using Zoom. Participant visits and contact points are detailed in Figure 1. The first counselling session will begin by discussing the PICF, answering any questions the participant may have, and gaining signed consent to continue with the study if the participant is happy to proceed. If counselling is being done by Zoom video conferencing, then the PICF will need to be signed and returned beforehand. Following this, the participant will be asked to complete a selection of validated questionnaires (see table 1). The purpose of these questionnaires is to capture the participant’s skin cancer knowledge, views and health behaviours prior to receiving genetic education and counselling. After completing the questionnaires, a genetic counsellor (or trained dermatologist) from the research team will conduct a pre-testing genetic education and counselling session that includes collection of personal and family medical history, education on genetics, the likelihood of detecting a mutation given the medical/family history and the possible impact of test results on the individual and their family members. At the end of this education and counselling session the participant will be asked again if they still wish to go ahead with the genetic testing, and if they do, a saliva sample will be requested. Participants will be asked if they would like to invite family members to join the study. If they do, then the genetic counsellor/dermatologist will identify specific family members that would meet the criteria for genetic testing, and therefore be eligible to join the study. Study brochures will be given to the participant to supply to family members. The visit will take between 1 to 1.5 hours in total.

In most cases the second counselling session for test reporting will occur approximately. 2-3 months after their first visit. This visit will either take place at the CRF- PAH or over online video conference call using Zoom, and will take approx. 1 to 1.5 hours. The session will begin by asking the participant to complete a questionnaire with selected validated instruments (see table 1). The purpose of these questionnaires is to measure any impact from the first genetic education and counselling appointment. Once questionnaires are complete, a genetic counsellor (or trained dermatologist) from the research team will conduct the post testing genetic counselling and reporting of genetic testing results. This process includes: test results, significance for personal risk, significance for family risk, recommended preventative health behaviour and screening. The genetic counsellor or dermatologist will offer to send a report of the genetic findings to the participant’s nominated healthcare provider. At the end of the counselling session, the participant will be asked if they consent to being contacted for a phone interview at a later date.

Post genetic testing, education and counselling questionnaires will be conducted at 2 weeks, 3 months and 12 months following reporting of test results. These questionnaires will be sent using the REDCap System, which facilitates an email containing a web-link to be sent for the participant to complete the questionnaire online, at their convenience. Two reminder emails to complete questionnaires will be sent out if not completed.

**Figure 1. A Flow chart of procedures involved for participants enrolled in Stage 1 of the study**

**Table 1: Questionnaires used at different time points.**

|  | Baseline (before counselling) | 2nd visit (before results) | 2nd visit (decliners) | 2 weeks | 3 months | 12 months |
| --- | --- | --- | --- | --- | --- | --- |
| **BACKGROUND INFORMATION** | | | | | | |
| Demographics | ✓ |  |  |  |  |  |
| Sun exposure | ✓ |  |  |  | ✓ | ✓ |
| Skin surveillance | ✓ |  |  |  | ✓ | ✓ |
| **HEALTH BELIEFS** | | | | | | |
| Risk perception^10, 21^ | ✓ | ✓ | ✓ | ✓ | ✓ | ✓ |
| Health Belief Model questionnaire^22, 23^ | ✓ |  |  |  |  | ✓ |
| Multidimensional Health Locus of Control (MHLC) Scale^24, 25^ | ✓ |  |  |  |  |  |
| **PSYCHOSOCIAL STATES AND TRAITS** | | | | | | |
| HADS (Hospital Anxiety and Depression Scale)^26^ | ✓ | ✓ | ✓ | ✓ | ✓ | ✓ |
| Cancer Worry Scale^27^ | ✓ |  |  |  | ✓ | ✓ |
| Fatalism Scale^28^ | ✓ |  |  |  | ✓ | ✓ |
| Belief in genetic determinism^29^ | ✓ |  |  |  |  |  |
| Monitor/Blunter scale^30, 31^ |  | ✓ | ✓ |  |  |  |
| State-Trait Anxiety Inventory (STAI)^32^ |  | ✓ | ✓ |  |  |  |
| **EXPERIENCE WITH GENETIC COUNSELLING AND TESTING** | | | | | | |
| Perceived benefits and limitations of genetic testing^10^ | ✓ |  |  |  |  |  |
| Genomic outcomes scale^33^ |  |  |  | ✓ |  |  |
| Genetic Counselling Satisfaction Scale (GCSS)^34^ |  | ✓ | ✓ | ✓ |  |  |
| Decision Regret Scale^35^ |  |  |  | ✓ |  | ✓ |
| Decision Satisfaction^36^ |  |  |  | ✓ |  |  |
| Perceived personal control (PPC)^37^ |  | ✓ | ✓ |  |  |  |
| MICRA (Multidimensional Impact of Cancer Risk Assessment)^38^ |  |  |  | ✓ | ✓ | ✓ |
| **DECLINING GENETIC TESTING** | | | | | | |
| Reasons for not testing^39, 40^ |  |  | ✓ |  |  |  |

*2.3.2. Semi-structured interviews (Stage 1, Cohort 1)*

A time and date convenient for the participant, will be organised to conduct the semi-structured interviews by phone call. The call will take approximately 30-60 minutes, and will take place approximately 1 month after genetic results were reported. At the start of the call, the researcher will check if the time/date is still convenient, and if it is not, they will reschedule. The interviews will be semi-structured, using a scripted guideline of questions. The interviews will be recorded using an audio recording device, with the participant’s permission.

*2.3.3. Cross-sectional Questionnaire (Stage 2, Cohort 2)*

This stage of the research will occur after all data is collected and analysed from Stage 1 of the research project. No study visit is required for Stage 2. We will use an online research platform, such as REDCap that will allow us to send an email to eligible participants, as described in recruitment (section 2.2.2). We will collect electronic consent for participation in Stage 2 of the study, before providing a link to the questionnaire to complete. This questionnaire can be completed at any time and it can be saved at any point to complete later. There will be no further follow up with participants in Stage 2, it will be a one-off (cross-sectional) questionnaire.

# **Genetic Counselling conducted by video conferencing (telegenetics)**

In line with measures for social distancing in response to the COVID-19 pandemic, we will now introduce an alternative to study visits at the Clinical Research Facility at PAH. For some participants we will conduct genetic counselling and education sessions via online video conferencing using Zoom (or similar), when possible. Prior to conducting the counselling session, a member of the research team will discuss the PICF in full over the phone with the participant and answer any questions. The participant will then need to sign the consent form and send it to us either by email or post. The counselling video-conferencing session will not occur until after the signed consent form has been received. After the counselling session, if the participant wishes to go ahead with testing, we will post them the self-collection saliva kit for genetic testing, with instructions on (pre-paid) postage for the sample.

A systematic review (Hilgart et al, 2012) of 12 separate studies has reported high levels of patient satisfaction with telegenetic services. Additionally when a comparison group was used, satisfaction levels were no different between those receiving face-to-face counselling.

# **Participants who decline genetic testing**

Some individuals may wish to take part in the research, but don’t wish to have genetic testing. Likewise, we expect that some participants may change their mind and decline genetic testing after receiving pre-testing genetic counselling and education, or decline receiving results after previously consenting to testing. We will ask these participants to remain in the study as they are valuable to our research in understanding barriers to genetic testing. For these participants, we will still provide genetic counselling and education based on their family history, rather than genetic testing (or not provide genetic test results if testing already completed). We would ask them to still complete pre and post questionnaires that measure their knowledge and perceptions on melanoma, psychological impact, and health behaviour. In addition we have a questionnaire designed to capture the reasons and motivation behind declining to genetic testing. We will also still invite them to take part in a semi-structured interview as part of Stage 1 of the research.

# **Dermatologist reporting genetic results**

Participants in Stage 1 of the study will be randomised to receive the genetic education, counselling and test reporting by either a genetic counsellor or a dermatologist (or dermatologically trained clinician). We will be using block randomisation, stratified by whether the participant is from a) CDKN2A positive family, or b) other familial melanoma mutation positive family, or c) no known melanoma mutation identified. Data linkage, descried in section five, will be used to identify the genetic status of past participants before randomisation to facilitate stratification.

The aim is to evaluate whether the person providing the genetic education and counselling (genetic counsellor or dermatologist), affects the psychosocial or behavioural outcomes. The dermatologists/clinician will be provided with training from genetic counsellors prior to providing genetic education, counselling and test reporting to participants. Questionnaire data collected throughout stage 1 of the study will be used to analyse any significant differences in psychosocial or behavioural outcomes.

## **Assessment of clinician training protocol**

To evaluate the success, and identify areas that require improvement, clinicians trained to provide genetic testing for this study will be interviewed. A semi-structured interview guide will be used to cover two topics, a) the training process, including the supporting documents and aids, and b) usefulness of genetic testing for familial melanoma. Audio recordings of the interviews will be transcribed verbatim, and used for thematic analysis following methods described in section 8.1.

- - 1. Recruitment of Clinicians for Interviews

To date (August 2021) there have been 8 clinicians trained to provide genetic test results. Any future trained clinicians may also be contacted about interview participation. Clinicians will be contacted via a short email, with the attached PICF. If no response to the email is received, then a follow up email will be sent 2 weeks later. A copy of standardised email recruitment text is provided in a separate document.

- - 1. Procedure for Clinician Interviews

Prior to interviews proceeding, Associate Investigator Clare Primiero will discuss the PICF with clinicians, answer any questions they have, and gain written consent if they are willing. Clare will then conduct the interviews at a time and place that is convenient for the Clinicians. If the clinician prefers to do the interview by phone, that will be arranged. If the clinician prefers to just answer the questions in text, rather then talking, then an email of the list of questions will be sent to the clinician. The clinician can then type their answers and email them back. Phone or in-person interviews will be audio recorded for later transcription. Transcription will be done by a professional contracted transcribing service.

- - 1. Sample Size and Analysis of Clinician Interviews

Interview transcripts will be used for qualitative analysis, using methods of thematic analysis (as described in section 8.1). The aim of qualitative research is to uncover a variety of opinions. There is no calculation used to determine sample size for qualitative research. The aim is to reach ‘saturation’ in new themes or perspectives found in the interview transcripts. For narrow research themes, such as the current study, saturation is often reached after around 10 participants. Our intention is to publish the results of the Clinician Interviews, and present at conferences. All clinicians trained in this study will be invited to co-author publications, regardless of whether they participate in the clinician interviews or not.

# **Data Linkage**

Many of the study participants in Stage 1 are expected to have already participated in a previous research study with our team (UQ DRC). In these instances, where participants have agreed, we plan to use data and samples collected from the previous research study, in the current study. This may include; questionnaire data and genetic sequencing results. Specifically, questionnaire data relating to health behaviour and sun protection will be used to produce longitudinal data when combined with the current study. The previous studies that participants may have been involved in with UQ-DRC are under Metro South HREC references; HREC/09/QPAH/162, HREC/16/QPAH/37, HREC/16/QPAH/125, and HREC/17/QPAH/816.

A separate consent page on the PICF explains the data linkage explicitly, and requires participants to separately consent to this aspect of the study.

# **Genetic Sequencing**

Saliva samples will be collected using an Oragene DNA self-collection kit (DNA Genotec), or similar. The sample will be sent to an NATA accredited genetic testing laboratory in the USA for panel testing of known genes associated with melanoma risk (Invitae Melanoma Panel). This process only involves looking at specific genes, and does not involve searching for novel genetic changes or markers in techniques such as Whole Exome Sequencing. Therefore, it is not possible to have any *incidental findings*, in which a gene mutation associated with an unrelated condition is found. As we are using a panel test that will cover multiple melanoma risk genes, it is therefore possible that a participant may be found to hold a second mutation that was not previously found in their family, and this may come as a surprise. This possibility is covered during the pre-testing genetic counselling and education. In the event that this occurs, appropriate genetic and/or psychological counselling will be provided (See Ethical Considerations section).

# **Data Analysis**

*7.1 Stage 1: Pre and post genetic counselling questionnaires*

All questionnaires used in Stage 1, are previously validated, with recommended protocols for quantitative analysis. In some cases, questionnaire responses will be pooled within previously defined groups (i.e. *CDKN2A* positive, other germline mutation positive, or no mutation found), and compared for significant statistical differences using paired t-tests or chi-square analysis. Differences between dermatologist and genetic counsellor reported results will also be analysed.

**8.1 Interview data**

Semi-structured interviews conducted in Stage 1 of this study and Clinician Interviews will be transcribed verbatim for thematic analysis. The objective of thematic analysis is to identify the themes within participants’ responses to interview questions. The process of thematic analysis has 6 distinct steps, which are described in Braun and Clarke’s (2006) publication on ‘Using thematic analysis in psychology’:

1. Familiarisation with the data: Read and re-read interview transcripts.
2. Generating initial codes to the data: A code is a brief description of what has been said. It highlights a section of data that is interesting or relevant to the research question. Some codes will be repeated throughout the dataset on different extracts, while others may only occur once.
3. Search for themes: group codes into themes and determine how the themes connect.
4. Review themes: Read all the extracts of coded data within a theme and determine whether they support the theme, or if they are better suited to another existing or new theme. Generate a thematic ‘map’ of analysis.
5. Defining and naming themes: Assign a descriptive name for each theme and define the overall story within that theme, quoting important extracts of coded data connected to that theme.
6. Producing the report: A final analysis of interview data in the context of study objectives and current literature. It is intended to publish the reported findings of the interview data in a peer-reviewed medical/science journal.

**8.2 Stage 2: Cross-sectional Questionnaire**

Statistical methods will be used to quantitatively analyse questionnaire data. Possible answers to questions will include binary answers (e.g. yes/no), or on a 5 point Likert Scale (e.g. strongly agree, agree, unsure, disagree and strongly disagree). Total and subscale scores will be computed and tested for normality. The statistical analysis plan will be further developed upon design of the final questionnaire.

# **Data Management**

Participant data will be handled with utmost confidentiality. A participant log will be kept where a coded ID number will be allocated to each participant. Interview transcriptions, questionnaire data, saliva samples, and genetic data will only have the participant ID number on them to protect privacy. The participant log will only be accessible to the study investigators. Electronic files are stored on the University of Queensland’s secured network, and password protected. Any hardcopy paper documents will be stored in locked cabinets within the card accessed TRI building.

Questionnaire data will be recorded on the REDCap system, which is a secured study database software developed by Vanderbilt University, and administrated by the Queensland Clinical Trials and Biostatistics Centre at the School of Public Health, The University of Queensland.

# **Dissemination of results**

Results from this study are planned to be disseminated through peer-reviewed publications, conferences and non-peer reviewed media outlets. No identifiable information will be used in publication of the results. As this study is being completed as part of Associate Investigator Clare Primiero’s PhD, results of this study will also be published in her thesis. No identifiable information will be used in the thesis.

We will generate a summary of research findings from Stage 1 and 2 of the study to be provided to our participants. This summary will be submitted to the HREC for approval before sending it out. The summary will be sent via email to participants who requested to receive this information on their PICF at the start of the study.

# **Ethical considerations**

The study will be reviewed by Metro South Health HREC located at Princess Alexandra Hospital, The University of Queensland HREC and the QIMR Berghofer Medical Research Institute HREC in accordance with the *National Statement of Ethical Conduct in Human Research (2007),* the International Guidelines known as *Good Clinical Practice* and the *World Medical Association’s Declaration of Helsinki.*

All participants will receive written and verbal information about the study. This information will emphasise that participation is voluntary and that they may withdraw from the study at any time for any reason without penalty or jeopardising their medical care. All participants will be given the opportunity to ask questions and will be given sufficient time to consider before consenting.

As the study involves reporting genetic test results, there is a risk of negative psychological reaction from participants. Although we believe a positive result for a genetic mutation inferring increased risk to melanoma would not be unexpected in this cohort, it could still be upsetting. Furthermore, some of the genes included in the testing panel are associated with risk for other cancers, and this may be unexpected. If this occurs, it will be discussed appropriately with the participant by a genetic counsellor. If the participant expresses significant upset, or if it is evident to any of the research team, the participant will be offered additional counselling. Participants are also provided with contact details to the study team if they think of further questions after their appointment, or if they’d like to see a genetic counsellor for an additional appointment. Any additional counselling appointments will be provided to participants at no charge to them.

# **References**

1. (AIHW) AIoHaW. Skin Cancer in Australia Canberra: AIHW; 2016 [Available from: [www.aihw.gov.au](file:///\\nas02.storage.uq.edu.au\shares\SOM-Dermatology\Common\Clare's%20Folder\PhD\Clinical%20Study\Ethics\HREC%20submission\www.aihw.gov.au).

2. Aitken JF, Duffy DL, Green A, et al. Heterogeneity of melanoma risk in families of melanoma patients. Am J Epidemiol. 1994;140(11):961-73.

3. Ransohoff KJ, Jaju PD, Tang JY, et al. Familial skin cancer syndromes: Increased melanoma risk. J Am Acad Dermatol. 2016;74(3):423-34; quiz 35-6.

4. Rossi M, Pellegrini C, Cardelli L, et al. Familial Melanoma: Diagnostic and Management Implications. Dermatology practical & conceptual. 2019;9(1):10-6.

5. Leachman SA, Lucero OM, Sampson JE, et al. Identification, genetic testing, and management of hereditary melanoma. Cancer Metastasis Rev. 2017;36(1):77-90.

6. Kasparian NA, Meiser B, Butow PN, et al. Predictors of psychological distress among individuals with a strong family history of malignant melanoma. Clin Genet. 2008;73(2):121-31.

7. Aoude LG, Wadt KA, Pritchard AL, et al. Genetics of familial melanoma: 20 years after CDKN2A. Pigment cell & melanoma research. 2015;28(2):148-60.

8. Halk AB, Potjer TP, Kukutsch NA, et al. Surveillance for familial melanoma: recommendations from a national centre of expertise. 2019;181(3):594-6.

9. Kefford RF, Mann GJ. Is there a role for genetic testing in patients with melanoma? Curr Opin Oncol. 2003;15(2):157-61.

10. Kasparian NA, Meiser B, Butow PN, et al. Genetic testing for melanoma risk: a prospective cohort study of uptake and outcomes among Australian families. Genet Med. 2009;11(4):265-78.

11. Aspinwall LG, Stump TK, Taber JM, et al. Genetic test reporting of CDKN2A provides informational and motivational benefits for managing melanoma risk. Transl Behav Med. 2018;8(1):29-43.

12. Aspinwall LG, Taber JM, Leaf SL, et al. Melanoma genetic counseling and test reporting improve screening adherence among unaffected carriers 2 years later. Cancer Epidemiol Biomarkers Prev. 2013;22(10):1687-97.

13. Glanz K, Volpicelli K, Kanetsky PA, et al. Melanoma genetic testing, counseling, and adherence to skin cancer prevention and detection behaviors. Cancer Epidemiol Biomarkers Prev. 2013;22(4):607-14.

14. Aspinwall LG, Leaf SL, Dola ER, et al. CDKN2A/p16 genetic test reporting improves early detection intentions and practices in high-risk melanoma families. Cancer Epidemiol Biomarkers Prev. 2008;17(6):1510-9.

15. Aspinwall LG, Taber JM, Kohlmann W, et al. Unaffected family members report improvements in daily routine sun protection 2 years following melanoma genetic testing. Genet Med. 2014;16(11):846-53.

16. Percival N, George A, Gyertson J, et al. The integration of BRCA testing into oncology clinics. Br J Nurs. 2016;25(12):690-4.

17. Slade I, Riddell D, Turnbull C, et al. Development of cancer genetic services in the UK: A national consultation. Genome Med. 2015;7(1):18.

18. Collins RE, Wright AJ, Marteau TM. Impact of communicating personalized genetic risk information on perceived control over the risk: a systematic review. Genet Med. 2011;13(4):273-7.

19. Claassen L, Henneman L, De Vet R, et al. Fatalistic responses to different types of genetic risk information: exploring the role of self-malleability. Psychol Health. 2010;25(2):183-96.

20. Taylor NJ, Mitra N, Qian L, et al. Estimating CDKN2A mutation carrier probability among global familial melanoma cases using GenoMELPREDICT. J Am Acad Dermatol. 2019;81(2):386-94.

21. Aspinwall LG, Stump TK, Taber JM, et al. Impact of melanoma genetic test reporting on perceived control over melanoma prevention. J Behav Med. 2015;38(5):754-65.

22. Champion VL. Instrument refinement for breast cancer screening behaviors. Nurs Res. 1993;42(3):139-43.

23. Champion VL. Instrument development for health belief model constructs. ANS Adv Nurs Sci. 1984;6(3):73-85.

24. Wallston KA, Wallston BS, DeVellis R. Development of the Multidimensional Health Locus of Control (MHLC) Scales. Health education monographs. 1978;6(2):160-70.

25. Ross TP, Ross LT, Short SD, et al. THE MULTIDIMENSIONAL HEALTH LOCUS OF CONTROL SCALE: PSYCHOMETRIC PROPERTIES AND FORM EQUIVALENCE. Psychol Rep. 2015;116(3):889-913.

26. Zigmond AS, Snaith RP. The hospital anxiety and depression scale. Acta Psychiatr Scand. 1983;67(6):361-70.

27. Custers JA, van den Berg SW, van Laarhoven HW, et al. The Cancer Worry Scale: detecting fear of recurrence in breast cancer survivors. Cancer Nurs. 2014;37(1):E44-50.

28. Shen L, Condit CM, Wright L. The psychometric property and validation of a fatalism scale. Psychol Health. 2009;24(5):597-613.

29. Carver RB, Castera J, Gericke N, et al. Young Adults' Belief in Genetic Determinism, and Knowledge and Attitudes towards Modern Genetics and Genomics: The PUGGS Questionnaire. PLoS One. 2017;12(1):e0169808.

30. Steptoe A. An abbreviated version of the Miller Behavioral Style Scale. Br J Clin Psychol. 1989;28 ( Pt 2):183-4.

31. Miller SM. Monitoring and blunting: validation of a questionnaire to assess styles of information seeking under threat. J Pers Soc Psychol. 1987;52(2):345-53.

32. Marteau TM, Bekker H. The development of a six-item short-form of the state scale of the Spielberger State-Trait Anxiety Inventory (STAI). Br J Clin Psychol. 1992;31 ( Pt 3):301-6.

33. Grant PE, Pampaka M, Payne K, et al. Developing a short-form of the Genetic Counselling Outcome Scale: The Genomics Outcome Scale. Eur J Med Genet. 2019;62(5):324-34.

34. DeMarco TA, Peshkin BN, Mars BD, et al. Patient satisfaction with cancer genetic counseling: a psychometric analysis of the Genetic Counseling Satisfaction Scale. Journal of genetic counseling. 2004;13(4):293-304.

35. Brehaut JC, O'Connor AM, Wood TJ, et al. Validation of a Decision Regret Scale. 2003;23(4):281-92.

36. Holmes-Rovner M, Kroll J, Schmitt N, et al. Patient satisfaction with health care decisions: the satisfaction with decision scale. Med Decis Making. 1996;16(1):58-64.

37. Berkenstadt M, Shiloh S, Barkai G, et al. Perceived personal control (PPC): a new concept in measuring outcome of genetic counseling. Am J Med Genet. 1999;82(1):53-9.

38. Cella D, Hughes C, Peterman A, et al. A brief assessment of concerns associated with genetic testing for cancer: the Multidimensional Impact of Cancer Risk Assessment (MICRA) questionnaire. Health Psychol. 2002;21(6):564-72.

39. Demyttenaere K, Evers-Kiebooms G, Decruyenaere M. Pitfalls in counseling for predictive testing in Huntington disease. Birth Defects Orig Artic Ser. 1992;28(1):105-11.

40. Surampalli A, Khare M, Kubrussi G, et al. Psychological Impact of Predictive Genetic Testing in VCP Inclusion Body Myopathy, Paget Disease of Bone and Frontotemporal Dementia. Journal of genetic counseling. 2015;24(5):842-50.
