# Supplementary material for: Protocol to evaluate a pilot program to upskill clinicians in providing genetic testing for familial melanoma
Source: PLoS One. 2022 Dec 7;17(12):e0275926. doi: 10.1371/journal.pone.0275926 (PMC9728910; doi:10.1371/journal.pone.0275926)
Supplement: S2 File — (PDF) [file pone.0275926.s005.pdf]

Participant Information and Consent Form For Clinician Interviews

Version: 2 dated 12/08/2021
Site: Clinical Research Facility, Princess Alexandra Hospital

---

Project Title: Investigation into the psychosocial impact of genetic testing for familial melanoma

Study: Clinician interviews to evaluate training process

Principal Investigator: Dr Aideen McInerney-Leo
Association Investigators: Clare Primiero, Betsy Peach, Dr Tatiane Yanes, Prof H. Peter Soyer, QIMR Berghofer Investigators: Prof Nick Hayward, Jane Palmer.

This Participant Information and Consent form is 6 pages long. Please make sure you have all the pages.

1. Your Consent

You have been invited to take part in this study because you have received training to provide genetic testing for familial melanoma, as part of our research study. The purpose of this study is to evaluate the training process you received and identify areas for improvement. A secondary aim of this study is to assess the usefulness of genetic testing for familial melanoma, from a clinician's point of view.

This study is being conducted at the Clinical Research Facility at Princess Alexandra Hospital (PAH) and is being led by Genetic Counsellor and Research Fellow, Dr Aideen McInerney-Leo from The University of Queensland.

This Participant Information Form contains detailed information about the research project and explains all the procedures involved. Knowing what is involved will help you decide if you wish to take part in the research. Please read this information carefully and ask any questions you may have about this study. Participation is entirely voluntary. If you don't wish to take part, you don't have to. Should you decide to take part in the research project, you will be asked to sign the Consent Form. By signing the Consent Form, you are telling us you:

- Understand the information;
- Consent to take part in the research project;

2. What is the background and purpose of this study?

The study titled "Investigation into the psychosocial impact of genetic testing for familial melanoma", which you have been involved in, is nearing completion. As part of this study, you were provided with training and supporting documents to provide genetic testing to participants for familial melanoma. Our aim is to improve our training protocol and supporting documents for future training initiatives. Your feedback is extremely valuable in this process.

3. What does participation in the research project involve?

Participation in this study involves a one-off interview which can be done either in person, over the phone, or by email exchange (i.e., a list of interview questions can be emailed to you, and you can then provide your typed response). The interviewee will use a semi-structured guide with questions covering two distinct topics. The first questions will ask for your thoughts on the training you received to provide genetic testing, and the supporting documents provided. The second topic will cover questions on the usefulness of genetic testing for familial melanoma. The interview will take on average about 30 minutes and will be audio recorded and transcribed for analysis.

4. Do I have to take part in this research project?

Participation in this research project is entirely voluntary. If you do not wish to take part, you do not have to. If you decide to take part and later change your mind, you are free to withdraw from the project at any stage.

Your decision whether to take part or not to take part, or to take part and then withdraw, will not affect your relationship with the study team or The University of Queensland.

5. What are the possible benefits of participating?

We aim to publish the results of this study in a scientific/medical journal, and present findings in conferences or seminars. Regardless of whether you participate in the interviews or not, we will invite you to be a co-author in any future publications on the results of this study. If you do not wish to be a co-author, you may still choose to review the manuscript prior to submission.

Benefits to the research and wider community may include improvement in future training initiatives for non-genetic specialists to provide clinical genetic testing.

6. What are the possible risks of participating in the study?

There are no foreseen risks involved in participating in this study.

7. What will happen to my information?

Audio recordings of the interview will be deleted after transcription is complete. Transcriptions of interviews will only be labelled with a study ID number, with no personal details that could identify you included. At the completion of the study, interview transcripts, along with all study documents are archived for 15 years, as per current guideline. Electronic files are password protected and stored on The University of Queensland's secured network. Any paper documents will be stored in locked cabinets within a card-accessed only building, located at the Translational Research Institute, PAH. After 15 years, electronic files can be deleted, and paper files are confidentially destroyed.

8. Privacy and Confidentiality

All interview transcripts will only contain a study ID number, which investigators can use to link back to your details if required. Interview transcripts of all participants are collated and used to analyse by finding recurring themes in participants' responses.

Our plan is to also publish our findings in scientific-medical journals and present our research at conferences and seminars. Any results published or presented will not contain your personal information. If you would like a copy of any reports published in journals, simply ask a member of the study team, we will be happy to share this with you.

9. What if I withdraw from this research project?

If you change your mind about participating in the exit interview at any stage before or during the interview, you can. Furthermore, at completion of the interview, if you wish to withdraw your consent for the audio recording of the interview to be used for analysis, you can. Please inform a member of the team if you wish to withdraw or stop the interview at any stage. Withdrawing will not affect your relationship with The University of Queensland.

10. Who is organising and funding the research?

This project is organised by researchers from The University of Queensland's Dermatology Research Centre. Funding for this project has come from a National Health and Medical Research Council Fellowship grant, an Australian Skin and Skin Cancer Centre Early Career Grant, and from an Australian Government Research and Training Scholarship.

11. Is this research project approved?

This project will be carried out according to the *National Statement on Ethical Conduct in Human Research* (2007) produced by the National Health and Medical Research Council of Australia. This statement has been developed to protect the interests of people who agree to participate in human research studies.

The ethical aspects of this research project have been reviewed by the Human Research Ethics Committee of Metro South Health and The University of Queensland.

12. Further information and complaints

If you require further information, or if you have any problems concerning this project you can contact the principal researcher;

Dr Aideen McInerney-Leo
Principal Investigator
Tel: 07 3443 7057
Email: a.mcinerney@uq.edu.au

If you have any complaints about any aspects of the project, the way it is being conducted or any questions about your rights as a research participant, then you may contact Metro South Health Human Research Ethics Coordinator;

HREC Coordinator
Tel: 07 3443 8047
Email: Ethicsresearch.PAH@health.qld.gov.au

Participant Information and Consent Form For Clinician Interviews

Version: 2 dated 12/08/2021
Site: Clinical Research Facility, Princess Alexandra Hospital

---

Project Title: Investigation into the psychosocial impact of genetic testing for familial melanoma

Study: Clinician interviews to evaluate training process

Principal Investigator: Dr Aideen McInerney-Leo
Association Investigators: Clare Primiero, Betsy Peach, Dr Tatiane Yanes, Prof H. Peter Soyer

---

- I have read the Participant Information Sheet or someone has read it to me in a language I understand.
- I understand the purposes, procedures and risks of the research described in the project.
- I have had an opportunity to ask questions and I am satisfied with the answers I have received.
- I freely agree to participate in this research project as described and understand that I am free to withdraw at any time during the project without affecting my future relationship with The University of Queensland.
- I understand that I will be given a copy of this document signed by me to keep.
- I may be approached again to participate in future components of this study or future studies conducted by the investigators, but I am under no obligation to do so.

Participant's Name (printed) ……………………………

Signature: ………………………………………………… Date: ………………………………

Email address:……………………………………………………

Would you like to receive a summary of our research findings? **Yes** ☐ **No** ☐

Declaration by researcher*: I have given an explanation of the research project, its procedures and risks and I believe that the participant has understood that explanation.

Researcher's Name (printed) …………………………………

Signature: ……………………………………………… Date: ………………………………

Revocation of Consent Form
For Clinician Interviews

Version: 2 dated 12/08/2021
Site: Clinical Research Facility, Princess Alexandra Hospital

---

Project Title: Investigation into the psychosocial impact of genetic testing for familial melanoma

Study: Clinician interviews to evaluate training process

Principal Investigator: Dr Aideen McInerney-Leo
Association Investigators: Clare Primiero, Betsy Peach, Dr Tatiane Yanes, Prof H. Peter Soyer

---

I hereby wish to **WITHDRAW** my consent to participate in the research project named above and understand that such a withdrawal WILL NOT jeopardise any aspect of my relationship with The University of Queensland or The Princess Alexandra Hospital.

Please tell us to which extent you would like to withdraw from the study. You can either consent to use information already collected, or request any information collected from you is destroyed. This is up to you.

☐ I no longer consent to the use of my (unidentifiable) information to be used for future research.

☐ I request that any information collected from or about me is destroyed and no longer used in this study or any future research.

Participant's Name (printed): ………………………………………………………..

Signature: ………………………………………….. Date: ……………………………..

Participant Information and Consent Form

Version: 2 dated 17/03/2020
Site: Clinical Research Facility, Princess Alexandra Hospital

---

Project Title: Investigation into the psychosocial impact of genetic testing for familial melanoma (Stage 1)

Principal Investigator: Dr Aideen McInerney-Leo
Association Investigators: Clare Primiero, Betsy Peach, Dr Tatiane Yanes, Prof H. Peter Soyer, QIMR Berghofer Investigators: Prof Nick Hayward, Jane Palmer.

This Participant Information and Consent form is 10 pages long. Please make sure you have all the pages.

1. Your Consent

You have been invited to take part in this study because you have been identified as a person with extensive personal and/or family history of melanoma. The purpose of this study is to investigate the effect genetic testing and counselling for familial melanoma has on individuals and their families. This study is being conducted at the Clinical Research Facility at Princess Alexandra Hospital (PAH), and is being led by Genetic Counsellor and Research Fellow, Dr Aideen McInerney-Leo from The University of Queensland.

This Participant Information Form contains detailed information about the research project and explains all the procedures involved. Knowing what is involved will help you decide if you wish to take part in the research.

Please read this information carefully and ask any questions you may have about this study. Before deciding whether or not to take part, you might want to talk about it with a relative, friend or your local doctor.

Participation is entirely voluntary. If you don't wish to take part, you don't have to.

Should you decide to take part in the research project, you will be asked to sign the Consent Form. By signing the Consent Form, you are telling us you:

- Understand the information;
- Consent to take part in the research project;
- Consent to have the tests that are described or you may decline this aspect of the study;
- Consent to the use of your personal and health information as described;

What is the background and purpose of this study?

Many people have a personal and/or family history of melanoma. In some families there is a gene which increases melanoma risk. Some of those genes have been identified and genetic testing is available. The purpose for genetic testing is to ensure people at increased risk are offered appropriate surveillance. Additionally, their family members can then be offered testing to see if they inherited the same genetic change. The purpose of this study is to evaluate whether genetic testing for melanoma in families with a strong history, affects people psychologically or affects their behaviour.

Worldwide it is estimated between 2-5% of melanoma cases are associated with an inherited gene mutation. The most common gene associated with familial melanoma is called the CDKN2A, which is found in 20 to 40% of the familial melanoma cases. Other much rarer genes associated with increased melanoma risk have been found, and are each responsible for less than 1% of the familial melanoma cases. Still, in at least half the cases of familial melanoma, no responsible gene mutation is found.

The purpose of this study is to investigate the psychological effect the genetic testing process has on individuals and their families.

2. What does participation in the research project involve?

Participation in this study involves;

- 1st session will include; Pre-test genetic counselling and education, completing questionnaires, and if you decide to have testing, collection of a saliva sample. This process will take about 1.5 hours. This will either be conducted in person at PAH, or conducted using online video conferencing.

- 2nd Session will include: Communication of genetic test results, provision of specific counselling depending on your results and completing further questionnaires. This appointment will also take about 1.5 hours, and will either be conducted online using video conferencing, or in person at the PAH

- 1 month after receiving your personalised genetic test results, counselling and education, we would like to phone you to ask questions surrounding your melanoma diagnosis experience and your involvement in the study so far. This will take about 30-60 minutes. This phone call will be recorded and transcribed to allow analysis of interviews.

- We will also email you questionnaires at 3 other time points for you to complete online, at your convenience. This will be at 2 weeks, 3 months and 12 months after receiving your genetic test results.

3. Genetic Counselling conducted by video conferencing.

In line with measures for social distancing in response to the COVID-19 pandemic, this study has introduced an alternative to site visits at the Clinical Research Facility at the PAH. When possible, we will conduct genetic counselling and education, and test reporting sessions using online video conferencing using a program called Zoom, which would allow us to email a link for the video conference to you. If you are using Zoom from your phone, you'll need to download the (free) Zoom application. We may need to use different video-conference applications, such as Skype. After your genetic counselling session, if you wish to go ahead with genetic testing, we will then post to you the self-collection saliva kit for genetic testing, along with instructions for (pre-paid) postage for your sample.

4. Will everyone receive genetic testing?

Not everyone who participates in this study will receive genetic testing. If you have been invited to join the study by a family member who is already a participant, then it depends on their genetic test results. If your family member was found negative for any known gene mutations, then genetic testing would not be offered to you. This is because it is very unlikely a known gene mutation associated with melanoma risk would be found in you or any members of your family. You will still receive counselling and education based on your family history, and your family member's genetic test result, from a genetic counsellor.

5. What if I don't want genetic testing?

If you don't want to have genetic testing, then we will not do any genetic testing. Likewise, to change your mind about having genetic testing after receiving the pre-testing genetic education and counselling, is perfectly okay. It is just as valuable to our research to collect questionnaire answers from people who have decided to decline genetic testing as those who have undertaken genetic testing. Therefore, if you would still like to be a part of our research, but not receive any genetic testing, then you will receive counselling and education regarding your melanoma risk based on your family history alone. We will still ask you to take part in a phone interview 1 month after your counselling and education session. Likewise, we would still ask you to complete online questionnaires at 2 weeks, 3 months and 12 months after your counselling and education appointment.

6. Do I have to take part in this research project?

Participation in this research project is entirely voluntary. If you do not wish to take part, you do not have to. If you decide to take part and later change your mind, you are free to withdraw from the project at any stage.

Your decision whether to take part or not to take part, or to take part and then withdraw, will not affect your rights in any way. You will still receive the same level of consideration and care and your standard treatment will not be affected.

7. What if I wish to withdraw from all or any part of this research project?

Please inform a member of the study team if you would like to withdraw from the research project. At the time of withdrawal, you can let us know to what extent you would like to withdraw from the study. You can either request to stop any future visits AND request we destroy any samples or information collected from you for the research project so far, OR you can just stop study visits but consent to the continued use of samples or information already collected for this research project.

8. What are the possible benefits of participating?

As part of the research project, you will receive education and counselling (with or without genetic testing) regarding your personal genetic risk for melanoma. Learning you have a genetic mutation that places you at increased risk of melanoma may be useful to you. Learning of your risk may assist you in planning appropriate sun protective behaviours and intervals for clinical skin examinations. This information may also be beneficial to your family.

Benefits to the research and wider community may include:

- Assisting researchers and clinicians to better understand the psychological impact the genetic testing and reporting of results has on people with familial melanoma
- Developing new and improved methods of genetic testing and reporting personal genetic risk results for familial melanoma.

9. What are the possible risks of participating in the study?

The overall risk is considered low, there are three possible results you could receive from this study:

1. You carry a mutation which increases your risk for melanoma. This may be in a familial melanoma gene or it may be in a familial cancer gene. Both can be associated with increased risk for other cancers. This will be discussed with you.
   - If a mutation is found in a familial cancer gene (e.g. hereditary breast cancer), this can be surprising, especially if there is no known family history. Follow up genetic counselling will be provided in these cases to ensure people have time to process the information, initiate a clinical surveillance plan and ask any questions they may have.

2. A known causative gene cannot be found in your family. This can be disappointing as you don't have an explanation for your family history.
3. You do not carry the mutation which has been found in other people in your family. The impact of this result affects people differently, however most are relieved by this result. .

Secondly, receiving genetic test results become part of your medical record and may possibly affect your ability to get certain insurances in the future, and may be used against you in the work setting. Additionally, this information can have implications for the health of your family members and could impact family relationships. All of this information will be addressed in the pre-test education and counselling session so you are in the best position to decide whether genetic testing is the right decision for you.

10. What will happen with my test samples and information about me?

Saliva samples collected from you will be sent to an accredited genetic testing laboratory (Invitae) in the USA to be tested for a panel of known genes associated with melanoma and other cancer risk. When results are entered into the study database for analysis, a code will be applied and identifying information removed. All other information collected in this study, such as questionnaires and interview transcripts will also only contain a study code and no personal details. Only the study investigators will be able to connect the code to your personal details when required, or have access to samples or information collected for this research. All electronic files are password protected and stored on the University of Queensland's secured network. Any paper documents will be stored in locked cabinets within a card-accessed only building, located at the Translational Research Institute, PAH.

11. What is the potential impact on my family if I take part?

The genes tested in this research study are typically inherited from one generation to the next. If you carry a mutation in the gene then your family members (parents, siblings and children), have a 50% chance of carrying the same mutation. In these instances, we recommend you invite family members who are at increased risk to consider genetic testing and/or counselling. As this study has implications for your family members, we also recommend before you decide to participate, you discuss this study with your family by sharing this information sheet. In this way, if mutations are found, then this information may not be as unexpected or upsetting to your family members.

12. Will I be given the results of the research project?

Part of this study involves returning your personal genetic test results back to you. As well, the combined results from our research, including questionnaire and interview data are pooled together for analysis. We plan to write a summary of our findings that can be sent to you if you wish to receive this update.

Our plan is to also publish our findings in scientific-medical journals, and present our research at conferences. Any results published or presented will not contain your personal information. If you would like a copy of any reports published in journals, simply ask a member of the study team, we will be happy to share this with you.

13. Will there be future research using my samples?

It is possible we may do other studies on genetic testing for melanoma in the future where it would be beneficial to compare these results with others. If you consent, we would like to reanalyse the information collected in the study for unspecified future research projects into skin cancer. If we had external collaborators, we would not disclose any information that could be used to identify you. Any future research projects will also first need to gain approval through a Human Research Ethics Committee. The option to consent to future use of your information is provided for you in the Consent Form at the end of this document. Your decision whether or not to consent to future use of your samples will not affect your eligibility to participate in this study.

14. Who is organising and funding the research?

This project is organised by researchers from The University of Queensland's Dermatology Research Centre. Funding for this project has come from a National Health and Medical Research Council Fellowship grant, an Australian Skin and Skin Cancer Centre Early Career Grant, and from an Australian Government Research and Training Scholarship.

15. Is this research project approved?

This research project has received ethical approval from the Human Research Ethics Committees of Metro South Health, The University of Queensland and QIMR Berghofer.

16. Will I get paid to participate in this study?

A $20 Coles/Myer voucher will be given to you at both site visits at the PAH, Clinical Research Facility.

17. Who can I contact if I have any questions or problems or complaints in relation to this study?

If you require further information, or if you have any problems concerning this project you can contact the principal researcher;

Dr Aideen McInerney-Leo
Principal Investigator
Tel: 07 3443 7057
Email: a.mcinerney@uq.edu.au

If you have any complaints about any aspects of the project, the way it is being conducted or any questions about your rights as a research participant, then you may contact Metro South Health Human Research Ethics Coordinator;

HREC Coordinator
Tel: 07 3443 8047
Email: Ethicsresearch.PAH@health.qld.gov.au

Participant Consent Form

Version: 1 dated 05/09/19
Site: Clinical Research Facility, Princess Alexandra Hospital

---

Project Title: Investigation into the psychosocial impact of genetic testing for familial melanoma (Stage 1)

Principal Investigator: Dr Aideen McInerney-Leo
Association Investigators: Clare Primiero, Betsy Peach, Dr Tatiane Yanes, Prof H. Peter Soyer

---

- I have read the Participant Information Sheet or someone has read it to me in a language I understand.
- I understand the purposes, procedures and risks of the research described in the project.
- I have had an opportunity to ask questions and I am satisfied with the answers I have received.
- I freely agree to participate in this research project as described and understand that I am free to withdraw at any time during the project without affecting my future health care.
- I understand that I will be given a copy of this document signed by me to keep.
- I may be approached again to participate in future components of this study or future studies conducted by the investigators but I am under no obligation to do so.
- I understand participation in this study does not replace or affect the normal care and discussions I may have or had with my treating doctors.

In respect to *future research use,* I give permission for the use of my DNA and questionnaire data to be used for the purpose of:

1. This research project only Yes ☐ No ☐
2. This research project and any future research projects in skin cancer Yes ☐ No ☐

Participant's Name (printed) .... Date of Birth: ....

Signature: .... Date: ....

Mailing address/Email address:..

Would you like to receive a summary of our research findings? Yes ☐ No ☐

Declaration by researcher*: I have given an explanation of the research project, its procedures and risks and I believe that the participant has understood that explanation.

Researcher's Name (printed) ....

Signature: .... Date: ....

Data Linkage Consent Form

Version: 1 dated 05/09/19
Site: Clinical Research Facility, Princess Alexandra Hospital

---

Project Title: Investigation into the psychosocial impact of genetic testing for familial melanoma (Stage 1)

Principal Investigator: Dr Aideen McInerney-Leo
Association Investigators: Clare Primiero, Betsy Peach, Dr Tatiane Yanes, Prof H. Peter Soyer

---

Some of our study participants have participated in a previous research study with our research group (The University of Queensland's Dermatology Research Centre). If part of the previous research study involved genetic sequencing and/or questionnaires relevant to your melanoma risk, then we would like to access this information to be used in the current project.

Please tick the below boxes if you consent to the following statements:

☐ I consent to the researchers of this study (Investigators named above), to request and have access to any **genetic sequencing results** relevant to melanoma risk, collected in previous research studies that I have participated in.

☐ I consent to the researchers of this study (Investigators names above), to request and have access to any **questionnaires completed** relevant to melanoma risk, collected in previous research studies that I have participated in.

Participant's Name (printed) ………………………………… Date of Birth: ………………………

Signature: ………………………………………………………… Date: …………………………………

Declaration by researcher*: I have given an explanation of the research project, its procedures and risks and I believe that the participant has understood that explanation.

Researcher's Name (printed) ………………………………………

Signature: ……………………………………………………… Date: …………………………………

Revocation of Consent Form

Version: 1 dated 05/09/19
Site: Clinical Research Facility, Princess Alexandra Hospital

---

Project Title: Investigation into the psychosocial impact of genetic testing for familial melanoma (Stage 1)

Principal Investigator: Dr Aideen McInerney-Leo
Association Investigators: Clare Primiero, Betsy Peach, Dr Tatiane Yanes, Prof H. Peter Soyer

---

I hereby wish to **WITHDRAW** my consent to participate in the research project named above and understand that such a withdrawal WILL NOT jeopardise any aspect of my care or treatment, or my relationship with The University of Queensland or The Princess Alexandra Hospital.

Please tell us to which extent you would like to withdraw from the study. You can withdraw from participating in any further research visits, while still consent to other aspects of our research to continue. This is up to you.

- ☐ I no longer wish to participate in research visits
- ☐ I no longer consent to the use of my (unidentifiable) information to be used for future research.
- ☐ I request that any information collected from or about me is destroyed and no longer used in this study or any future research.

Participant's Name (printed): ………………………………………………………..

Date of Birth: ……………………………

Signature: ………………………………………….. Date: …………………………..
